# Members of the RhoA subfamily of GTPases suppress the stimulatory effect of the HPV E6 oncoprotein on the ubiquitin ligase E6AP

**DOI:** 10.1038/s41598-026-67837-8

**Published:** 2026-08-25

**Authors:** Daniela Eichbichler, Jasmin Jansen, Nadja Eulich, Florian Stengel, Martin Scheffner

**Affiliations:** 1 Department of Biology, University of Konstanz, Konstanz, Germany; 2 Konstanz Research School Chemical Biology, University of Konstanz, Konstanz, Germany

**Keywords:** E6AP/UBE3A, RhoA, E6 oncoprotein, Human papillomavirus, Angelman syndrome, Structural mass spectrometry, Cancer, Cell biology, Molecular biology, Oncology

## Abstract

The ubiquitin ligase E6AP, encoded by the *UBE3A* gene, plays a prominent role in human papillomavirus (HPV)-induced cancers. In complex with the HPV E6 oncoprotein, it targets inter alia the tumor suppressor p53 for degradation, thereby contributing to cellular transformation. Besides affecting the substrate spectrum of E6AP, E6 acts as a potent activator of the catalytic activity of E6AP. Here we report that RhoA and its subfamily members RhoB and RhoC serve as substrates of the E6-E6AP complex, but more strikingly, also act as strong inhibitors of the complex. We show that RhoA subfamily members potently inhibit E6-E6AP-mediated ubiquitination and degradation of p53 and that this inhibitory effect is not due to simple substrate competition. In addition to establishing RhoA subfamily members as the first known cellular regulators of the E6-E6AP complex, we provide evidence that RhoA binds to E6AP in the absence of E6. Notably, loss of functional E6AP is the cause of Angelman syndrome (AS), a neurodevelopmental disorder, and RhoA plays a critical role in maintaining synaptic structure and function. Thus, we propose that the interaction of RhoA subfamily members with E6AP is of significance for the development of both HPV-induced cancers and AS.

**Supplementary Information:**

The online version contains supplementary material available at 10.1038/s41598-026-67837-8.

## Introduction

Deregulation of the E3 ubiquitin-protein ligase E6AP has been associated with the development of several human disorders. E6AP was originally identified by virtue of its interaction with the E6 oncoprotein of so-called high-risk types of human papillomaviruses (HPVs) that *inter alia* cause cervical cancer and oropharyngeal cancer^1–3^. Furthermore, there is evidence to indicate that in addition to HPV-induced cancers, E6AP may also be involved in the development of other types of cancer^4^. Besides tumorigenesis, deregulation of E6AP plays a critical role in the development of distinct neurodevelopmental disorders. Genetic alterations of the E6AP-encoding gene *UBE3A* resulting in loss of E6AP expression or in the expression of E6AP variants with compromised E3 activity are the cause of the Angelman syndrome (AS)^5–8^, whereas amplification of the chromosomal region (15q11-13) that contains the *UBE3A* gene^9,10^ is found in individuals with Dup15q syndrome^11–14^.

Despite its critical role in human health and disease, only little is known about the cellular processes and pathways that are controlled by E6AP. To delineate these, identification of substrate proteins of E6AP appears to be most relevant. For cervical cancer, several substrates of the E6-E6AP complex including the tumor suppressor p53 and several PDZ domain-containing proteins have been identified^15,16^; yet the list of substrates may not be complete. Similarly, a number of proteins have been reported to serve as potential substrates for E6AP also in the absence of E6, including HHR23A/HHR23B, Ring1B, Ephexin-5, BK channels, PRMT5, and TKT1^17–22^. However, the actual importance of E6AP-mediated ubiquitination/degradation of these proteins for AS and/or Dup15q syndrome remains mostly unclear.

Similar to the identification of substrate proteins, it is important to elucidate the processes and mechanisms that regulate E6AP activity. Notably, the paternal *UBE3A* allele is silenced in certain brain areas by a long non-coding RNA^23–25^, and the functional maternal allele is lost in approximately 75% of AS individuals resulting in significantly reduced E6AP levels^7,8^. On the contrary, individuals with Dup15q syndrome harbor an additional *UBE3A* allele^13,14^ potentially resulting in increased E6AP levels. In addition, the *UBE3A* gene encodes three isoforms that differ in their very N-terminal regions^10^ and their subcellular localization^26^. While the differences in subcellular localization imply that the isoforms are in part involved in different cellular processes, it remains unclear if the isoforms differ in their biochemical/functional properties. Finally, there is evidence that the catalytic activity of E6AP is subject to regulation. The phosphorylation status of T485 (numbering according to human isoform 1) was reported to affect E6AP activity^27^. Furthermore, we reported that E6AP can be allosterically activated by the HPV E6 oncoprotein^28^ and by HERC2^29^, a member of the HECT E3 family that has been causally associated with a neurodevelopmental syndrome with AS-like features^30^, as well as by small molecules^31,32^. The notion that with respect to catalytic activity, E6AP adopts distinct conformational states has recently been proven by studies determining the structure of E6AP in complex with the HPV E6 oncoprotein and p53^33–35^.

In an attempt to identify potential substrates of the E6-E6AP complex at a proteome-wide scale, we recently identified the small GTPase RhoA as possible target^36^. RhoA caught our attention, because (1) it plays a crucial role in the regulation of the cytoskeleton and cell polarity^37,38^, i.e. structures/processes of importance for HPVs as they infect and replicate in stratifying epithelia^39,40^, and (2) *Ube3a*-deficient mice display increased RhoA activity and reduced dendritic spine density^19^ which is discussed to be a consequence of increased RhoA activity. Furthermore, *Ube3a*-deficient neurons were reported to have deficits in cytoskeletal dynamics^41^, and knockdown of E6AP expression in SH-SY5Y cells (neuroblastoma-derived cell line) has distinct effects on the maturation of focal adhesions^42^. Thus, we looked more closely into the potential interaction of RhoA, RhoB, and RhoC (i.e. members of the RhoA subfamily of Rho GTPases) with the E6-E6AP complex and E6AP alone. We report that the RhoA subfamily members may indeed represent substrates of E6-E6AP, but moreover, act as potent inhibitors of E6-facilitated ubiquitination of p53 as well as of other substrates of the E6-E6AP complex. The inhibitory effect of the RhoA subfamily members is not due to simple substrate competition, as RhoA and p53 do not compete for binding to E6-E6AP and RhoA does not need to be ubiquitinated to exert its inhibitory effect. We further provide evidence that whereas both E6 and E6AP are required for robust interaction with RhoA, E6AP represents the primary interaction partner for RhoA. That is, E6AP, but not E6, binds to RhoA in the absence of the respective other, though with low affinity. Thus, RhoA subfamily members are the first known cellular negative regulators of the ubiquitin ligase activity of the E6-E6AP complex and by directly interacting with E6AP, may be involved in E6AP-related processes in general.

## Results

### RhoA subfamily members interfere with E6-stimulated auto-ubiquitination of E6AP

**Fig. 1:**
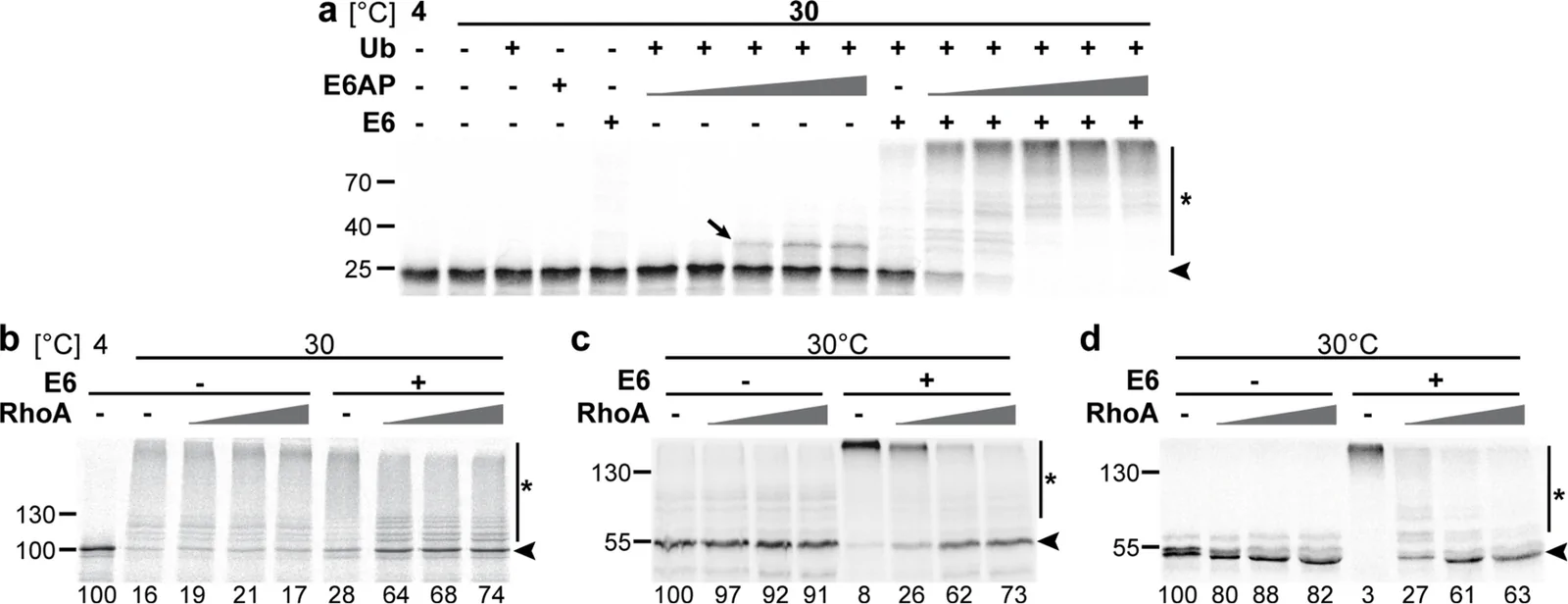
RhoA blocks the E3 activity of the E6-E6AP complex in vitro. **a**, RhoA is ubiquitinated by E6-E6AP in vitro. In vitro translated, radiolabeled RhoA was incubated with increasing concentrations of recombinant E6AP and the E6 oncoprotein under standard ubiquitination conditions (“Methods”) as indicated. After 90 min at 30 °C, whole reaction mixtures were analyzed by SDS-PAGE followed by fluorography. Running positions of molecular mass markers (kDa), unmodified RhoA (arrowhead) and ubiquitinated forms of RhoA (asterisk) are indicated. A presumably mono-ubiquitinated form of RhoA in the presence of E6AP is marked by an arrow. **b-d**, RhoA blocks the E3 activity of the E6-E6AP complex. In vitro translated, radiolabeled E6AP (**b**), p53 (**c**), and NHERF1 (**d**) were incubated with recombinant E6AP and increasing concentrations of GST-RhoA in the absence or presence of E6 under standard ubiquitination conditions as indicated. After 90 min at 30 °C, whole reaction mixtures were analyzed by SDS-PAGE followed by fluorography. Relative levels of non-modified E6AP/p53/NHERF1 were quantified with levels in the absence of both E6 and RhoA set to 100 and are indicated below the respective panel. Running positions of molecular mass markers (kDa), unmodified E6AP/p53/NHERF1 (arrowhead), and ubiquitinated forms of E6AP/p53/NHERF1 (asterisk) are indicated. The experiments shown are representative of three independent experiments.

In a recent proteomics study^36^, we identified RhoA and RhoC as potential substrates of E6AP in complex with the E6 oncoprotein of HPV-16 (referred to as “E6” in the following). E6 not only utilizes E6AP to target proteins like p53 that in the absence of E6 do not serve as substrates for E6AP, but also acts as strong allosteric activator of E6AP^28^. We therefore asked whether RhoA and RhoC are ubiquitinated in vitro by the E6-E6AP complex and by E6AP alone. Furthermore, we included the third member of the RhoA subfamily of Rho GTPases, RhoB in the analysis (RhoB was not identified in the proteomics study, but is highly similar to RhoA and RhoC at the amino acid sequence level; see Supplementary Fig. 1). Ubiquitination assays with in rabbit reticulocyte lysate (RL) translated, radiolabeled RhoA, RhoB, and RhoC showed that they are efficiently ubiquitinated by the E6-E6AP complex, but not or only slightly by E6AP alone (Fig. 1a, Supplementary Fig. 1). This validates the results of the proteomics study^36^ and suggests that similar to p53, RhoA subfamily members are bona fide substrates of E6-E6AP, but presumably not of E6AP alone.

To obtain more insight into the requirements of E6-E6AP-mediated ubiquitination of RhoA subfamily members (as the assays above were performed with Rho proteins translated in RL, proteins in addition to the basic components of the ubiquitin conjugation machinery may be involved), we performed ubiquitination assays with all components expressed in bacteria (Supplementary Fig. 2a-c). This again showed that RhoA subfamily members represent substrates for E6-E6AP in vitro, but not for E6AP alone. Unexpectedly, however, we also realized that in the presence of both E6 and RhoA subfamily members, less ubiquitin was consumed by E6AP during the reaction than in the absence of E6. Under the conditions used, E6AP ubiquitinates itself (“auto-ubiquitination”), a property that is stimulated by E6^43,44^. We therefore reasoned that the decreased consumption of ubiquitin by E6AP in the presence of both E6 and RhoA is explained by the notion that RhoA somehow interferes with E6-stimulated auto-ubiquitination of E6AP. To prove this assumption, we performed auto-ubiquitination assays with radiolabeled E6AP. Indeed, the presence of RhoA blocked auto-ubiquitination of E6AP, but only in the presence of E6 (Fig. 1b). Thus, the inhibitory effect of RhoA is specific, insofar as it does not interfere with the E3 activity of E6AP in general.

In the following, we refer to RhoA only, as RhoA subfamily members are highly similar at the amino acid sequence level (approximately 85–90% identity; Supplementary Fig. 1) and the results obtained are generally similar for RhoA, RhoB, and RhoC.

### RhoA interferes with E6-E6AP-mediated ubiquitination of p53

We next asked whether the inhibitory effect of RhoA is limited to E6AP auto-ubiquitination or whether it also interferes with ubiquitination of established substrates of the E6-E6AP complex. To do so, we determined the effect of RhoA on E6-E6AP-mediated ubiquitination of p53^45^ (Fig. 1c, Supplementary Fig. 2e) and the PDZ domain-containing protein NHERF1^46^ (Fig. 1d). This unambiguously showed that RhoA interferes with ubiquitination of both proteins in a dose-dependent manner. Importantly, Rac1 and Cdc42, two GTPases that are closely related to the RhoA subfamily members^47^ (Supplementary Fig. 1a) did not affect E6-E6AP-mediated ubiquitination of p53 (Supplementary Fig. 2e), indicating the specificity of the inhibitory effect of RhoA (note that Rac1 and Cdc42 are ubiquitinated by E6-E6AP in vitro, though less efficiently than RhoA subfamily members; Supplementary Fig. 1).

**Fig. 2:**
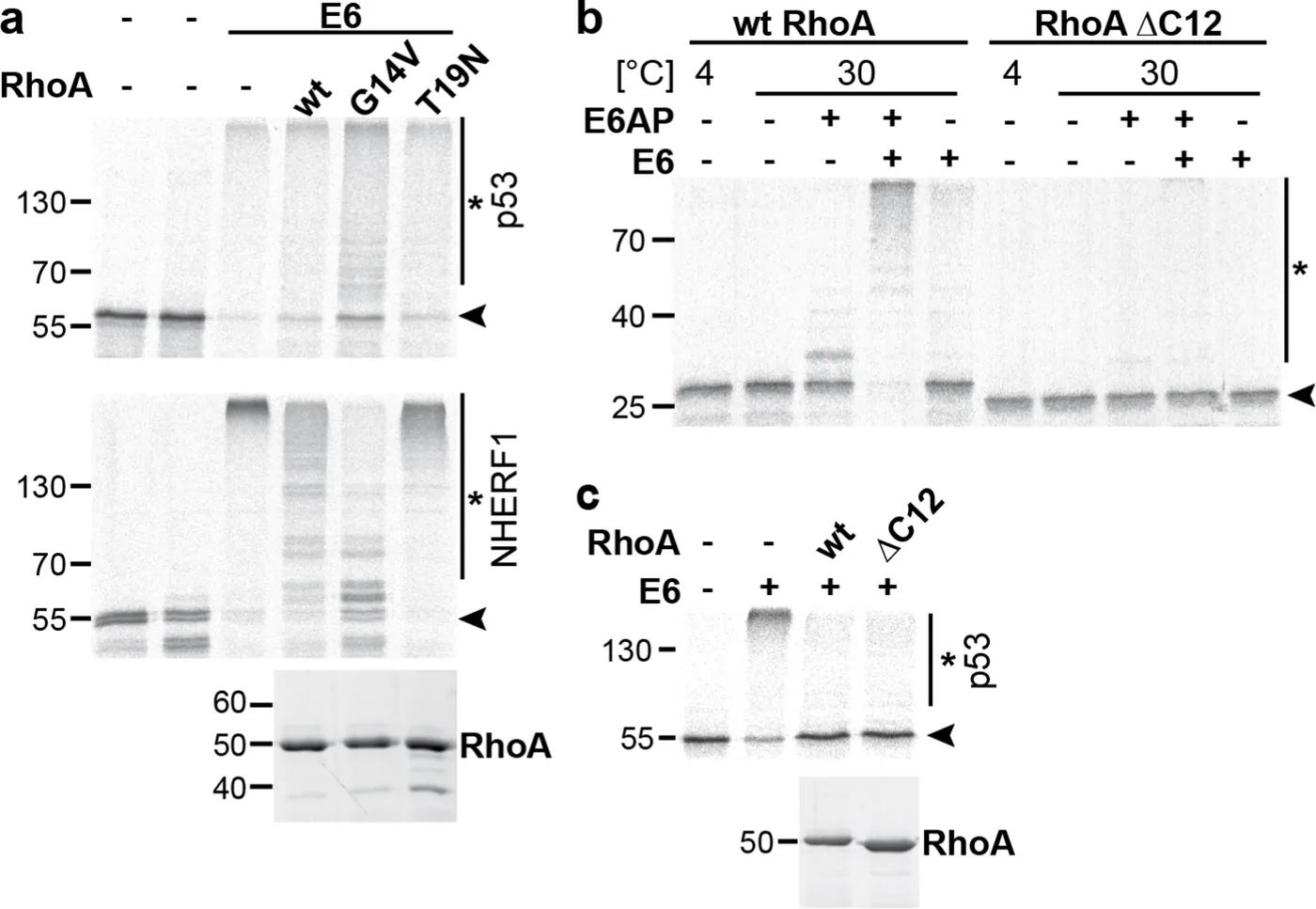
GTP binding facilitates RhoA-mediated inhibition of the E6-E6AP complex. **a**, The GTP-bound form of RhoA inhibits E6-E6AP-mediated ubiqutination in vitro. In vitro translated, radiolabeled p53 (upper panel) or NHERF1 (middle panel) were incubated with recombinant E6AP and the E6 oncoprotein in the absence or presence of wild-type RhoA (wt), RhoA G14V (constitutively active variant, which selectively binds GTP), and RhoA T19N (constitutively inactive variant, which selectively binds GDP) under ubiquitination conditions (“Methods”). After 90 min at 30 °C, reaction mixtures were analyzed by SDS-PAGE followed by fluorography. Running positions of molecular mass markers (kDa), unmodified p53/NHERF1 (arrowhead), and ubiquitinated forms of p53/NHERF1 (asterisk) are indicated. Lower panel shows input levels of GST-tagged wt RhoA, RhoA G14V, and RhoA T19N monitored by SDS-PAGE followed by Coomassie staining. Note that to clearly show that RhoA G14V is more efficient in inhibiting E6-E6AP than wt RhoA - representing a mixture of RhoA-GDP (inactive) and RhoA-GTP (active) in unknown ratio -, we employed RhoA amounts that are not sufficient to fully interfere with E6-E6AP-mediated ubiquitination. **b**, The C-terminal region of RhoA is required for E6-E6AP-mediated ubiquitination. In vitro translated, radiolabeled RhoA (wt) or the C-terminally truncated variant RhoA ΔC12 (deletion of the C-terminal 12 amino acids) were incubated with recombinant E6AP and the E6 oncoprotein under ubiquitination conditions (“Methods”) as indicated. After 90 min at 30 °C, reaction mixtures were analyzed by SDS-PAGE followed by fluorography. Running positions of molecular mass markers (kDa), unmodified wt RhoA/RhoA ΔC12 (arrowhead), and ubiquitinated forms of wt RhoA (asterisk) are indicated. **c**, Ubiquitination of RhoA is not required for inhibition of E6-E6AP-mediated ubiquitination. In vitro translated, radiolabeled p53 (upper panel) was incubated with recombinant E6AP and E6 in the absence or presence of RhoA (wt) or RhoA ΔC12 as indicated. After 90 min at 30 °C, reaction mixtures were analyzed by SDS-PAGE followed by fluorography. Running positions of molecular mass markers (kDa), unmodified p53 (arrowhead), and ubiquitinated forms of p53 (asterisk) are indicated. Lower panel shows input levels of GST-tagged RhoA and RhoA ΔC12 monitored by SDS-PAGE followed by Coomassie staining. The experiments shown are representative of three independent experiments.

So far, we have used in vitro translated and bacterially expressed RhoA without knowing its GDP/GTP-binding status; i.e. they likely consisted of a mixture of RhoA-GDP and RhoA-GTP, which represent the inactive form and the active form of RhoA, respectively, in unknown ratio. A convenient possibility to generate RhoA variants resembling the GTP-bound state or the GDP-bound state is the use of the single point mutants RhoA G14V and RhoA T19N that mimic the active state and the inactive state, respectively^38,48,49^. In vitro ubiquitination assays showed that RhoA G14V is most efficient in interfering with the E3 activity of E6-E6AP (Fig. 2a, Supplementary Fig. 3), indicating that it is mainly the active, i.e. GTP-bound form that exerts the inhibitory activity. We also determined the effect of RhoA G14V on E6AP-mediated ubiquitination of Ring1b. Ring1b represents a substrate of E6AP alone (i.e. in the absence of E6)^18^, but its ubiquitination is stimulated by the additional presence of E6^28^. Similar to E6AP auto-ubiquitination (Fig. 1b), RhoA inhibited E6-E6AP-mediated ubiquitination of Ring1b, but had no effect on E6AP-mediated ubiquitination (Supplementary Fig. 3a, 3b). This supports the notion that the inhibitory effect of RhoA is specific for the E6-E6AP complex, at least under the conditions used (see “Discussion”).

### The inhibitory effect of RhoA on E6-E6AP-mediated ubiquitination of p53 is not due to substrate competition

We previously reported that the presence of p53, i.e. a substrate protein, interferes with E6-stimulated auto-ubiquitination of E6AP^43^ or in other words, the E3 activity of E6AP is shifted from auto-ubiquitination towards ubiquitination of p53, representing a special case of substrate competition. A similar scenario can be envisioned for RhoA-mediated inhibition of E6-facilitated auto-ubiquitination of E6AP. To determine if the inhibitory effect of RhoA on E6-E6AP-mediated ubiquitination of p53 and other substrate proteins is also based on “simple” substrate competition, we first studied if ubiquitination of RhoA is at all required for it to exert its inhibitory effect. Mass spectrometric analysis of in vitro ubiquitinated RhoA revealed that a number of lysine residues distributed over the entire RhoA sequence are modified by E6-E6AP (Supplementary Fig. 3c). Yet, subsequent mutational analysis clearly showed that the lysine residues at the very C terminus of the Rho proteins are crucial for efficient ubiquitination (Supplementary Fig. 3d-f and data not shown). Accordingly, a respective deletion mutant of RhoA (RhoA ΔC12) is not or inefficiently ubiquitinated by E6-E6AP (Fig. 2b). Importantly, RhoA ΔC12 still interfered with E6-E6AP-mediated ubiquitination of p53 in vitro (Fig. 2c).

**Fig. 3:**
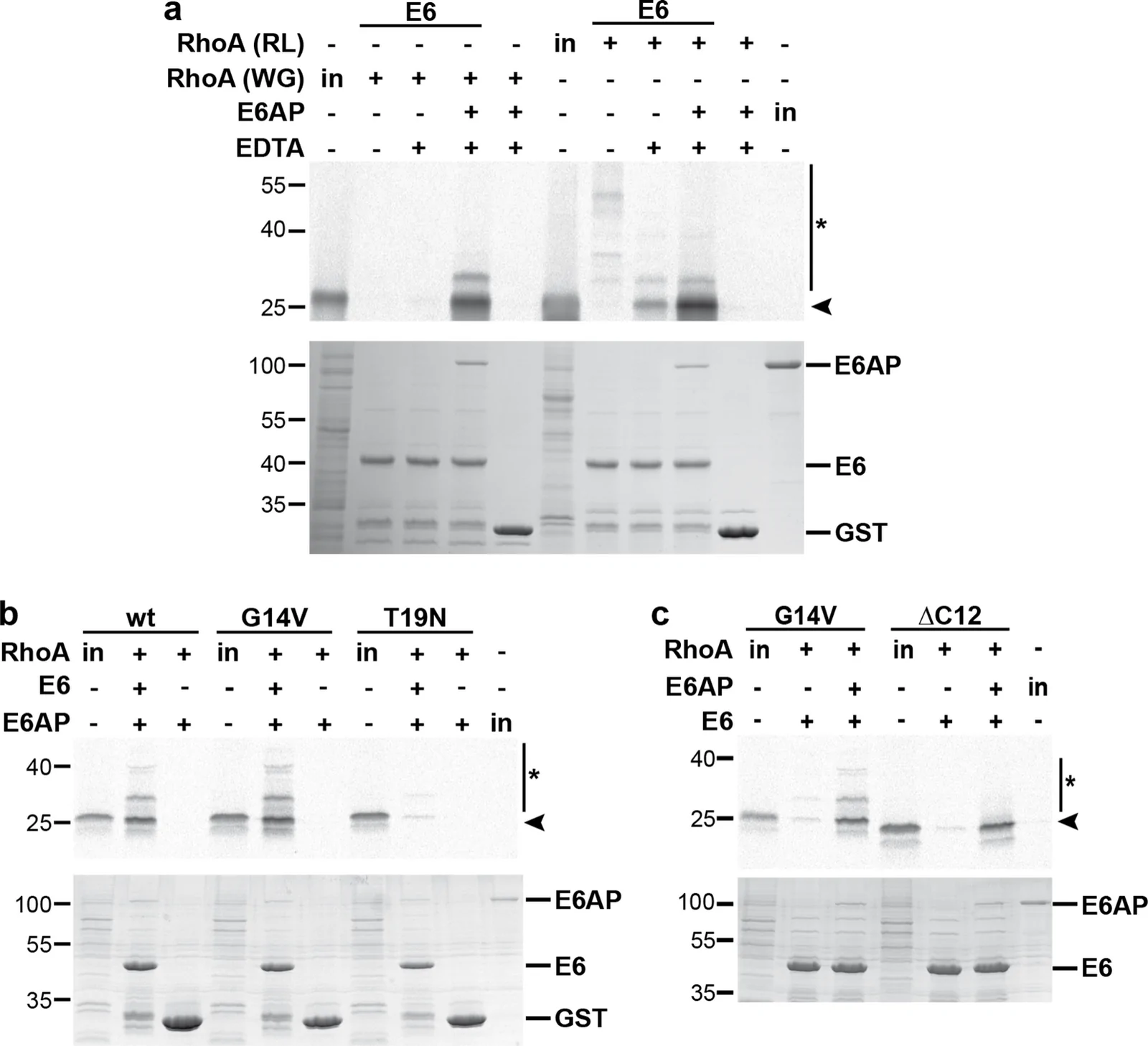
The GTP-binding status of RhoA affects its ability to form a ternary complex with E6 and E6AP. **a**, RhoA forms a ternary complex with E6 and E6AP. GST-tagged E6 (E6) bound to glutathione sepharose (GSH) beads was incubated with in vitro translated, radiolabeled RhoA in the absence and presence of recombinant E6AP and 20 mM EDTA (to block ubiquitination) as indicated. As control, GSH beads decorated with GST alone were used. RhoA was translated in wheat germ extract (WG) or in rabbit reticulocyte lysate (RL), since in contrast to RL, WG does not contain endogenous E6AP^1^. After 90 min at 4 °C, beads were washed and bound proteins were analyzed by SDS-PAGE followed by Coomassie blue staining (lower panel) to monitor levels of GST-E6, GST, and E6AP, and subsequently by fluorography (upper panel) to detect radiolabeled RhoA. Running positions of molecular mass markers (kDa), unmodified RhoA (arrowhead), ubiquitinated forms of RhoA (asterisk), GST-E6, GST, and E6AP are indicated. Input (in), 10% of in vitro translated RhoA and 100% of E6AP used in the binding reaction. **b**, Ternary complex formation depends on the GTP binding status of RhoA. Binding reactions were performed as in **a** with in vitro translated, radiolabeled RhoA (wt), RhoA G14V (mimicking the active, GTP-bound form of RhoA), or RhoA T19N (mimicking the inactive, GDP-bound form). Bound proteins were analyzed by SDS-PAGE followed by Coomassie staining (lower panel) to monitor levels of GST-E6, GST, and E6AP, and subsequently by fluorography (upper panel) to detect radiolabeled RhoA. Running positions of molecular mass markers (kDa), unmodified RhoA variants (arrowhead), ubiquitinated forms of RhoA variants (asterisk), GST-E6, GST, and E6AP are indicated. Input (in), 10% of in vitro translated RhoA variants and 100% of E6AP used in the binding reaction. **c**, RhoA ΔC12 forms a ternary complex with E6 and E6AP. Binding reactions were performed as in **a** with in vitro translated, radiolabeled RhoA G14V or RhoA ΔC12. Bound proteins were analyzed by SDS-PAGE followed by Coomassie blue staining (lower panel) to monitor levels of GST-E6 and E6AP and subsequently by fluorography (upper panel) to detect the radiolabeled RhoA variants. Running positions of molecular mass markers (kDa), unmodified RhoA variants (arrowhead), ubiquitinated forms of RhoA G14V (asterisk), GST-E6, and E6AP are indicated. Input (in), 10% of in vitro translated RhoA variants and 100% of E6AP used in the binding reaction. The experiments shown are representative of three independent experiments.

While the above data show that the inhibitory effect of RhoA does not require its ubiquitination, they do not exclude the possibility that RhoA and p53 compete for binding to E6-E6AP. To address this, we first determined whether RhoA forms a stable complex with E6-E6AP in conventional coprecipitation assays. We incubated a GST fusion protein of E6 with in vitro translated, radiolabeled RhoA in the absence and presence of bacterially expressed E6AP. In addition, for in vitro translation we used either RL or wheat germ extract (WG), as WG does not contain endogenous E6AP, while RL does^1^. This clearly showed that RhoA binds to E6 in a detectable manner only in the additional presence of E6AP (Fig. 3a). Note that to show that the ability of RhoA to bind to E6-E6AP does not require its prior ubiquitination, EDTA - which blocks the first step of the ubiquitination cascade - had to be added, as otherwise RhoA was ubiquitinated by E6-E6AP during incubation (see also Supplementary Fig. 4). In line with the observed inhibitory potential (Fig. 2a), a stable interaction was observed with RhoA G14V but not with RhoA T19N (Fig. 3b). In addition, RhoA ΔC12 was found to stably interact with E6-E6AP (Fig. 3c). These data support the notion that to efficiently interfere with the E3 activity of the E6-E6AP complex, RhoA has to stably interact with E6-E6AP, but it does not require its ubiquitination.

**Fig. 4:**
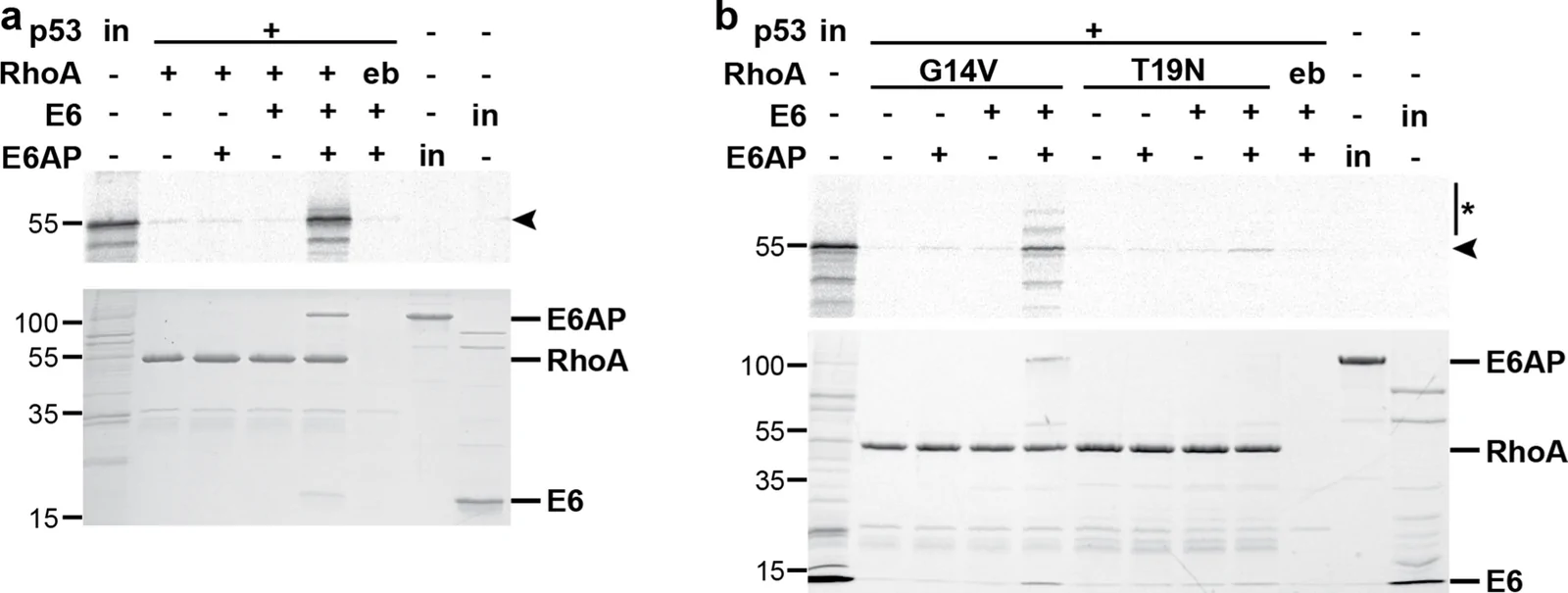
RhoA forms a quaternary complex with E6, E6AP, and p53. **a**, RhoA does not compete with p53 for binding to E6-E6AP. GST-tagged RhoA bound to GSH beads (RhoA) or empty GSH beads (eb) were incubated with in vitro translated, radiolabeled p53 in the absence and presence of recombinant E6AP and E6 (untagged form) as indicated. After 90 min at 4 °C, beads were washed, and bound proteins were analyzed by SDS-PAGE followed by Coomassie blue staining (lower panel) to monitor levels of GST-RhoA, E6AP, and E6, and subsequently by fluorography (upper panel) to detect radiolabeled p53. Running positions of molecular mass markers (kDa), p53 (arrowhead), GST-RhoA, E6AP, and E6 are indicated. Input (in), 10% of in vitro translated p53 and 100% of E6AP and E6 used in the binding reaction. **b**, The ability of RhoA to form a quaternary complex with E6, E6AP, and p53 depends on the GTP-binding status. Binding reactions were performed as described in **a** with GST-tagged RhoA G14V and GST-tagged RhoA T19N or empty GSH beads (eb) as control. Bound proteins were analyzed by SDS-PAGE followed by Coomassie blue staining (lower panel) to monitor levels of GST-RhoA variants, E6AP, and E6, and subsequently by fluorography (upper panel) to detect radiolabeled p53. Running positions of molecular mass markers (kDa), p53 (arrowhead), GST-RhoA variants, E6AP, and E6 are indicated. Input (in), 10% of in vitro translated p53 and 100% of E6AP and E6 used in the binding reaction. The experiments shown are representative of three independent experiments.

Next, we performed coprecipitation experiments with GST-tagged RhoA and in vitro translated, radiolabeled p53 in the absence or presence of bacterially expressed versions of untagged E6 and/or His-tagged E6AP. Remarkably, a stable interaction of p53 with RhoA was observed when both E6 and E6AP were present (Fig. 4a). This demonstrates that RhoA and p53 do not compete for binding to E6-E6AP, but rather form a quaternary complex with these. In addition, a stable interaction of E6AP with RhoA was only observed in the presence of E6, and formation of a quaternary complex was strongly affected by the GDP/GTP-binding status of RhoA (Fig. 4b).

In conclusion, the ability of RhoA to interfere with E6-E6AP-mediated ubiquitination of p53 (and other substrates) is not explained by mere substrate competition.

### E6AP represents the primary interaction partner for RhoA

The finding that for stable interaction with RhoA, both E6 and E6AP are required is reminiscent of the situation with p53^1,45^. Structural characterization of the p53-E6-E6AP complex showed that E6 represents the primary interaction partner of p53 and that the interaction with E6AP drives E6 into a conformation that is more competent in p53 binding^50^. We previously reported that cross-linking coupled to mass spectrometry (XL-MS) is suited to study the conformational dynamics of the interaction of E6AP with E6 as well as with small molecules that stimulate the E3 activity of E6AP^31,32,51^. Thus, we used XL-MS to determine if RhoA affects the conformational dynamics of the E6-E6AP complex (note that we used RhoA G14V in the following, as the active, i.e. GTP-bound form interferes with E6-E6AP activity most efficiently). Using the bifunctional amine-selective crosslinker DSS (disuccinimidyl suberate), the presence of RhoA resulted in a decline in the number of crosslinks, mainly within E6AP, for unknown reasons (Supplementary Fig. 5). Yet importantly, in the presence of both E6 and E6AP, two crosslinks between E6AP and RhoA were reliably detected (Table 1), while no crosslinks could be observed between E6 and RhoA.

**Table 1:** XL-MS analysis of the E6-E6AP-RhoA interaction suggests that RhoA binds directly to E6AP.

E6AP + E6 1 µM + 1.5 µM		E6AP + RhoA 1 µM + 2.5 µM		E6AP + E6 + RhoA 1 µM + 1.5 µM + 2.5 µM		E6 + RhoA 1.5 µM + 2.5 µM	
DSS							
*E6AP*	*E6*	*E6AP*	*RhoA*	*E6AP*	*RhoA*	*E6*	*RhoA*
K7	K72	---	---	K7	K135	---	---
K799	K65			K148	K162		
K799	K68						
K799	K72			*E6*	*RhoA*		
				---	---		
SDA							
*E6AP*	*E6*	*E6AP*	*RhoA*	*E6AP*	*RhoA*	*E6*	*RhoA*
G20	K121	Y104	F39	E185	K135	---	---
L293	K121	T106	Y34	V267	S188		
P498	Y70	K109	V38				
E752	K121	E185	K135	*E6*	*RhoA*		
		I265	S188	S71	D67		
		E269	S188				
		L781	T163				
		K847	D65				

List with confidently identified interlinks between the proteins indicated using DSS (upper part) or SDA (lower part) as crosslinker. For E6AP, numbering is according to human isoform 1^10^. For further details, see Methods.

Although interpretation of the data obtained with DSS may be difficult (i.e. although unlikely, due to the low overall number of crosslinks detected in the presence of RhoA, we may have missed additional existing crosslinks), they suggest that RhoA directly contacts E6AP (rather than E6). To substantiate this hypothesis, we resorted to XL-MS with SDA (succinimidyl 4,4’-azipentanoate) as crosslinker. SDA links lysine residues (by means of succinimide coupling to amines) with almost any other amino acid residues (via diazirine-based photoreaction chemistry). The data obtained by XL-MS with SDA clearly confirm that an interaction between E6 and RhoA can only be observed in the presence of E6AP. Moreover, they also indicate that E6AP and RhoA can interact with each other in the absence of E6 (Table 1, Supplementary Fig. 5).

Encouraged by the XL-MS data and to support the notion that E6AP can interact with RhoA also in the absence of E6, we switched to a method (spectral shift analysis; for details, see “Methods”) that allows to monitor interactions that are not amenable to conventional coprecipitation analyses. In brief, a fluorescently labeled GST fusion protein of RhoA was incubated with increasing concentrations of E6AP and/or E6, and potential interactions measured by means of a spectral shift from 670 nm to 650 nm (for more details, see “Methods”). The results obtained support the XL-MS data insofar as an interaction between E6AP and RhoA with a Kd in the low µM range was observed, while an interaction of E6 and RhoA could not be detected (Table 2, Supplementary Fig. 6). Remarkably, the presence of both E6 and E6AP increased the affinity for RhoA by approximately two orders of magnitude. Based on these and the XL-MS data, we conclude that E6AP represents the primary interaction partner for RhoA and that E6 stabilizes a RhoA-binding competent conformation of E6AP.

**Table 2:** The interaction of RhoA with E6AP is stabilized by the E6 oncoprotein.

	K_d_ (confidence interval)		
**G14V + E6AP + E6** 0.05µM + 2.45µM + 8.4µM	**32.3 nM** (17.0 nM – 61.5 nM)	**27.6 nM** (11.5 nM - 66.2 nM)	**32.4 nM** (19.9 nM – 52.9 nM)
**G14V + E6AP** 0.05µM + 4.9µM	**1.17 µM** (0.42 µM – 3.25 µM)	**2.63 µM** (0.79 µM – 8.73 µM)	**4.18 µM** (1.08 µM – 16.10 µM)
**G14V + E6** 0.05µM + 8.4µM	No interaction detectable		

Spectral shift analysis of fluorescently labeled GST-RhoA G14V in the presence of E6 or E6AP or both as indicated. For each condition, the results of three replicates are shown. E6AP and E6 were added to RhoA in serial dilutions up to 4.9 µM and 8.4 µM, respectively. For the analysis of the ternary complex, concentrations of RhoA G14V and E6AP were fixed as indicated, and E6 was added in serial dilution up to 8.4 µM.

**Fig. 5:**
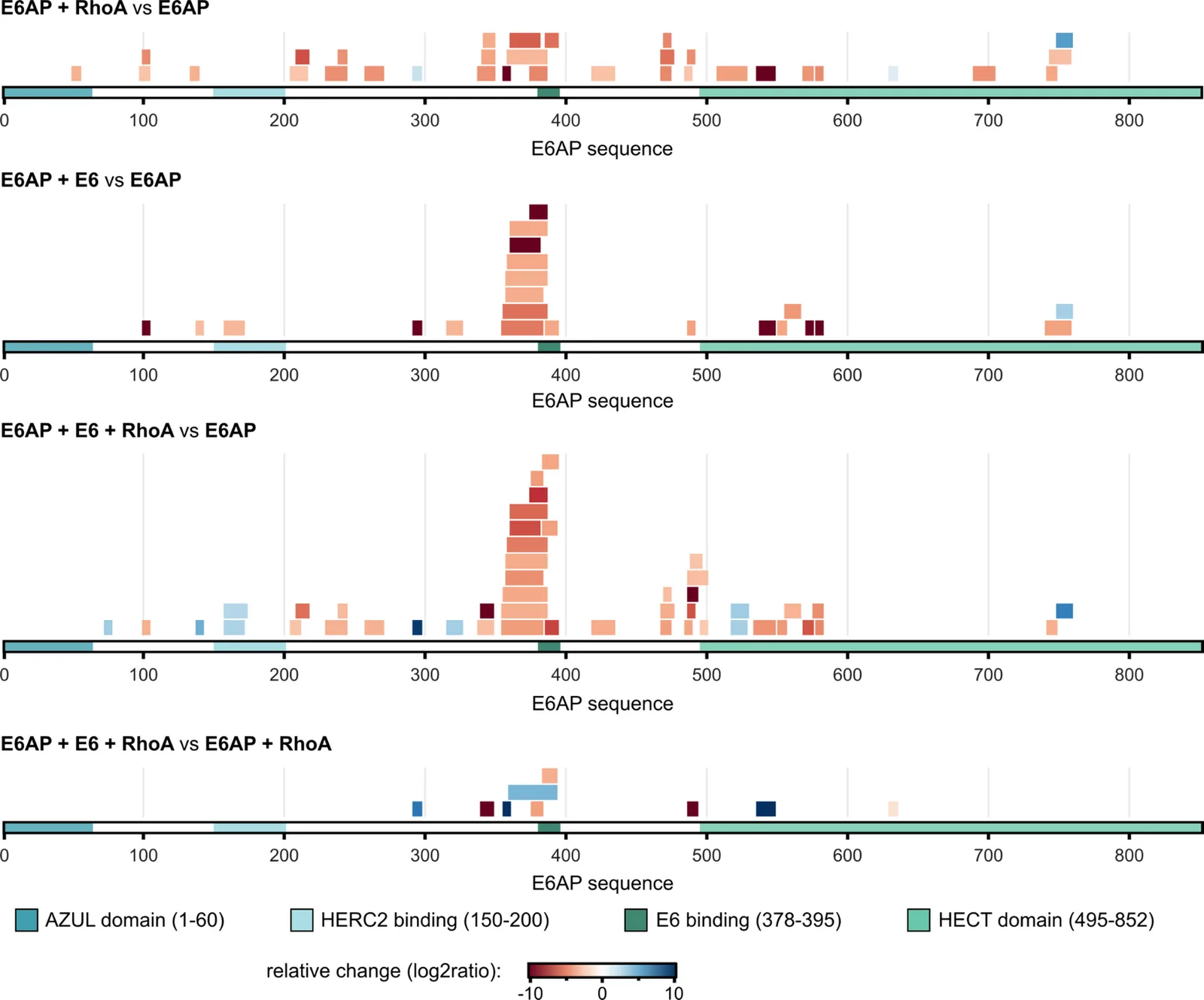
Binding of RhoA and of the HPV E6 oncoprotein affect similar regions of E6AP. Shown are significantly altered peptide quantities of E6AP using LiP-MS in the presence of RhoA or E6 or of both. Differential analysis of peptide quantities was performed with the MSstatsLiP package^81^, and hits were considered significant with adjusted p-value < 0.05 and log2ratio > 1 (indicated in blue) or <-1 (indicated in red). For comparisons, E6AP alone or E6AP in the presence of RhoA were used as reference as indicated. For further details, see “Methods”.

To obtain first information about the region(s) of E6AP that may directly contact RhoA, we employed limited proteolysis coupled to mass spectrometry (LiP-MS)^52,53^. The rationale for doing so is that by binding to E6AP, RhoA alters the pattern of peptides obtained by proteolytic digest by affecting the conformation and/or by protecting the region(s) of E6AP, to which RhoA binds, from the attack of proteases; i.e. RhoA leaves a “footprint” on E6AP. Indeed, as we previously reported^32^, in presence of E6 a region of E6AP that comprises the primary interaction site for E6 (amino acids 378–395)^54^ is protected from proteolytic digest (Fig. 5). Surprisingly, in the presence of RhoA, we observed a peptide pattern similar to the one obtained with E6, though the peptide quantities were somewhat lower than with E6. However, this is expected, as the interaction of RhoA with E6AP is significantly less robust than the E6-E6AP interaction (Table 1). In addition, in the presence of both E6 and RhoA, the protection of the region of E6AP spanning from approximately amino acid 350 to 500 was further enhanced, though only slightly. Taken together, the data suggest that E6 and RhoA bind to neighboring, or slightly overlapping regions of E6AP, a hypothesis that is supported by the fact that the additional presence of E6 does not significantly alter the peptide pattern of E6AP obtained with RhoA.

### RhoA interferes with the E3 activity of the E6-E6AP complex in cells

**Fig. 6:**
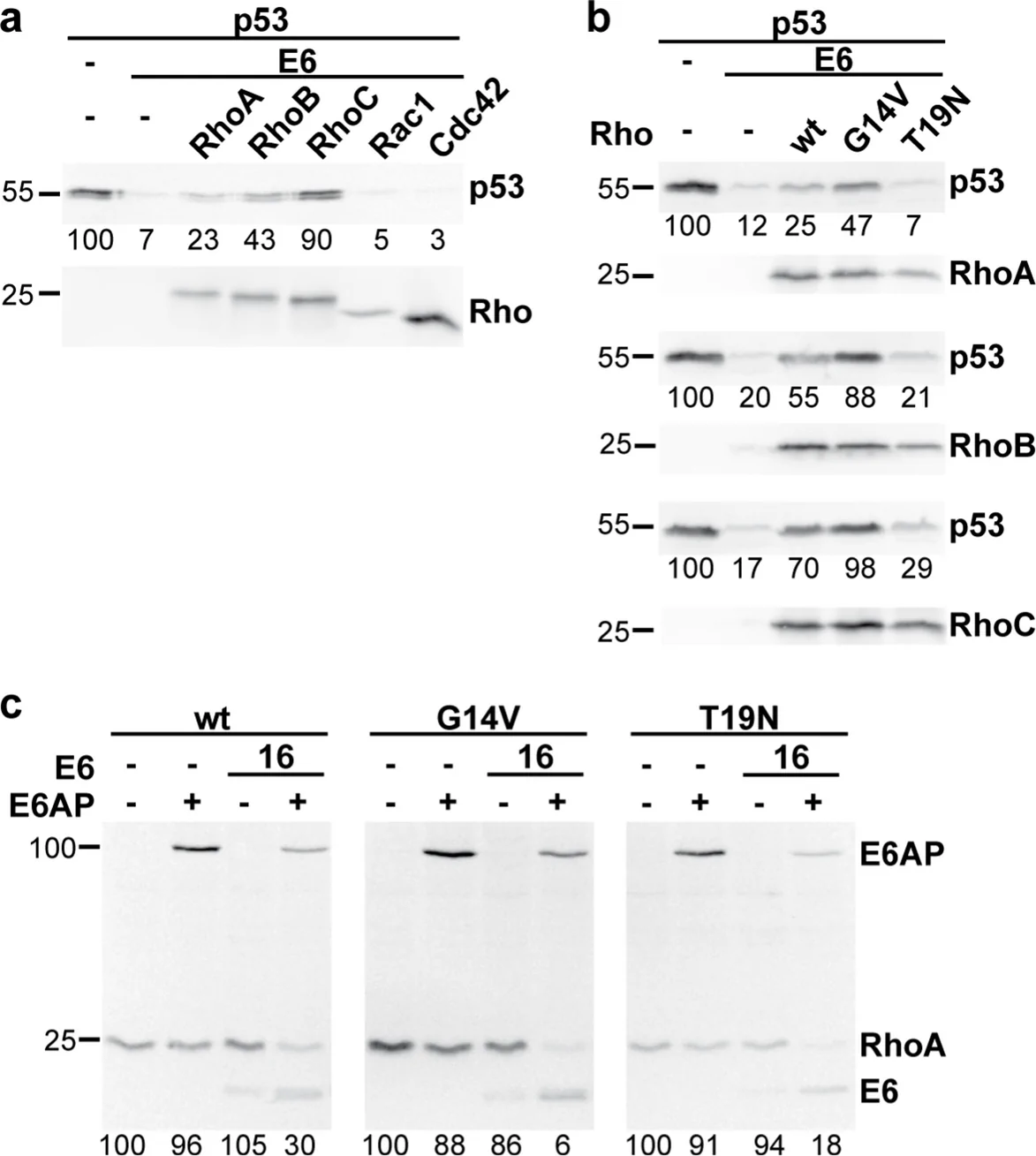
RhoA blocks the E3 activity of the E6-E6AP complex in cells. H1299 cells were transfected (**a**) with expression constructs encoding HA-tagged forms of p53, E6, RhoA, RhoB, RhoC, Rac1, and Cdc42, and (**b**) with expression constructs encoding HA-tagged forms of p53, E6, wild-type (wt) RhoA, RhoB, RhoC, and respective constitutively active (G14V) and inactive (T19N) variants as indicated. 24 h post-transfection, cell lysates were prepared, and transfection efficiency adjusted aliquots (“Methods”) were subjected to SDS-PAGE followed by western blot analysis. Expression levels of RhoA subfamily members and their variants as well as the closely related Rho GTPases Rac1 and Cdc42 were monitored by an anti-HA antibody; p53 was detected by an anti-p53 antibody. Running positions of molecular mass markers (kDa) are indicated. Note that under the conditions used, levels of HA-tagged E6 were too low to be detected by western blot. **c**, Overexpression of E6AP targets RhoA for degradation in an E6-dependent manner. H1299 cells were transfected with expression constructs encoding HA-tagged forms of E6AP, E6, wt RhoA, RhoA G14V, and RhoA T19N as indicated. 24 h post-transfection, cell lysates were prepared, and transfection efficiency adjusted aliquots (“Methods”) were subjected to SDS-PAGE followed by western blot analysis using an anti-HA antibody. Running positions of molecular mass markers (kDa), E6AP, E6, and RhoA and its variants are indicated. Relative levels of p53 (a, b) and RhoA (c) were quantified with levels in the absence of E6 set to 100 and are indicated below the respective panel. Note that in contrast to p53 degradation, E6AP has to be overexpressed (i.e. endogenous E6AP is not sufficient) and higher amounts of E6 are required to observe RhoA degradation. This indicates that under the conditions used in **a** and **b**, RhoA does not represent a substrate for the E6-E6AP complex. Furthermore, as reported^85^, coexpression of E6AP results in increased levels of E6. The experiments shown are representative of three independent experiments.

Since RhoA interferes with the E3 activity of the E6-E6AP complex, we were wondering if ectopic expression of RhoA or activation of endogenous RhoA interferes with the viability of HPV-positive cervical cancer cell lines such as HeLa^55^ and SiHa^56^. While ectopic expression of RhoA clearly affected the viability of HeLa and SiHa cells, this effect was not specific insofar as the viability of HPV-negative cells (e.g. H1299 cells) was similarly affected (data not shown). This result was not unexpected though, as RhoA plays a pivotal role in the regulation of several pathways that are crucial for cellular proliferation/viability irrespective of the HPV status of a cell. Thus, to obtain preliminary evidence that RhoA can act as an inhibitor of E6-E6AP in cells, we performed cotransfection experiments with H1299 cells that express E6AP but not p53^57,58^. As expected, expression of E6 resulted in efficient degradation of ectopically expressed p53 (Fig. 6). In line with the results obtained in vitro, coexpression of RhoA interfered with E6-induced degradation of p53 and NHERF1, whereas coexpression of Rac1 and Cdc42 had no effect (Fig. 6a, Supplementary Fig. 7a and 7b). Moreover, RhoA G14V was most efficient in inhibiting p53 degradation, indicating that also in cells, it is mainly the active, GTP-bound form that exerts the inhibitory activity (Fig. 6b).

Since RhoA is ubiquitinated by E6-E6AP in vitro, we also looked into the possibility that RhoA can serve as a substrate for E6-E6AP also in cells. Indeed, levels of RhoA, RhoA G14V, and RhoA T19N are significantly decreased in the presence of ectopically expressed E6 and E6AP (Fig. 6c). Since this decrease in levels is observed only in the presence of catalytically active E6AP (Supplementary Fig. 7c), we conclude that RhoA represents a potential substrate for E6-E6AP in vitro and in cells and that this property of RhoA seems to be less dependent on the GTP binding status than its inhibitory potential. Furthermore, in contrast to p53, endogenous E6AP levels are not sufficient to support E6-mediated degradation of RhoA (i.e. it has to be ectopically overexpressed). This indicates that under the conditions used to study p53 degradation (Fig. 6a and b), RhoA does not represent a substrate for E6-E6AP and, thus, that similar to the situation in vitro, the inhibitory effect of RhoA is not due to substrate competition.

## Discussion

It is commonly accepted that that the “hostile takeover” of the E3 ubiquitin ligase E6AP by the viral E6 oncoprotein plays a crucial role in HPV-induced carcinogenesis and presumably in the viral life cycle as well^59,60^. It is, therefore, of considerable interest to obtain a comprehensive overview on the substrate spectra of the E6-E6AP complex and of E6AP alone (i.e. in HPV-negative cells) as well as on the mechanisms that regulate their activity. Here, we report that the RhoA subfamily members do not only represent potential substrates of the E6-E6AP complex, but moreover interfere with its E3 activity by directly interacting with E6AP. Thus, RhoA subfamily members represent the first known natural inhibitors of the E6-E6AP complex.

Similar to p53, RhoA subfamily members bind with high affinity to the E6-E6AP complex. In contrast to p53^50^, however, E6AP represents the primary interaction partner, rather than E6. Of note, our LiP-MS data indicate that E6 and RhoA affect similar regions of E6AP comprising the primary interaction site for E6^50,54^ (Fig. 5). Yet, since E6 and RhoA bind simultaneously to E6AP, the primary binding sites of RhoA and E6 must be different. This indicates that the binding site of RhoA is close to the E6-binding site and/or that the effect of RhoA on the E6-binding site is due to RhoA-induced conformational rearrangements rather than direct physical interaction with it. We previously reported that E6 acts as an allosteric activator of E6AP by affecting the structural dynamics of E6AP such that E6AP-catalyzed isopeptide bond formation between ubiquitin and a substrate’s lysine residue is stimulated^28,51^. Since binding of RhoA almost completely inhibits the E3 activity of the E6-E6AP complex, RhoA stabilizes a conformation or conformational ensemble of E6AP that is still competent for E6 binding, but catalytically inactive (i.e. the “conformational” effect of RhoA is dominant over that of E6).

Both full-length RhoA and a C-terminal truncation mutant devoid of the so-called CAAX motif act as inhibitors of the E6-E6AP complex, but only the full-length protein represents a potential substrate (i.e. the two properties of RhoA can be separated; Fig. 2). In cells, the C-terminal CAAX motif (Supplementary Fig. 1a) is proteolytically processed upon lipidation of the C residue, thereby determining the location of RhoA at the plasma membrane^61^. This raises the questions as to whether and where the E6-E6AP complex (or E6AP alone) and RhoA actually meet each other in cells. Firstly, E6AP exists in three isoforms that differ in their very N-terminal extensions^10^. While the shortest isoform, isoform 1, has been reported to localize mainly to the nucleus, the other isoforms are mostly cytoplasmic^26,62^. Moreover, the intracellular localization of E6AP appears to change during neural development^63^. Importantly, the three isoforms behave similar with respect to both RhoA ubiquitination and RhoA-mediated inhibition (data not shown). Secondly, similar to E6AP, E6 is found in the nucleus and the cytoplasm^64^. Thirdly, RhoA can also reside in the nucleus^65,66^. Taken together, the data available indicate that the endogenous E6-E6AP complex (or E6AP alone) have the potential to interact with endogenous RhoA in cells, which is also supported by the results of our cotransfection assays (Fig. 6). Yet, determining the actual cellular localization, where this interaction takes place, as well as obtaining insight into the physiological consequences of this interaction represent considerable challenges.

A particular challenge in elucidating the physiological consequences of the E6-E6AP-RhoA interaction is that RhoA (and RhoA subfamily members) can be both substrate and regulator of the E6-E6AP complex. Concerning the former, it is generally difficult to prove that a protein of interest represents a physiologically relevant substrate in cells, mainly because substrate proteins are often targeted by several E3s rather than just by one. For instance, RhoA has been reported to be a substrate of the ubiquitin ligases Smurf1^67^, making it difficult to provide robust evidence that endogenous E6-E6AP are involved in RhoA degradation. Furthermore, E6-E6AP and RhoA may target each other only at certain stages during viral infection and/or during HPV-induced carcinogenesis. For instance, RhoA and/or the other subfamily members may interfere with the E3 activity of the E6-E6AP complex in early stages of infection as part of a host defense mechanism. Also, there is evidence to suggest that RhoA mainly acts as an oncoprotein, while RhoB has tumor-suppressive properties^68,69^. Thus, RhoB may be the physiological relevant substrate of the E6-E6AP complex, while conversely, RhoB may attempt to inhibit E6-E6AP-mediated ubiquitination of p53 for instance. The ability to separate the property of the Rho proteins to serve as substrates from their property to act as inhibitors of the E6-E6AP complex (Fig. 2) may eventually prove helpful to address the physiological significance of this potential mutual regulation.

In this study, we focused on the E6 oncoprotein of HPV-16, mainly because HPV-16 is the most prevalent HPV type found in cervical cancer^70^ and the interaction of E6AP with 16 E6 is well characterized at the biochemical and structural level. To determine, if the ability of 16 E6 to interact with RhoA in the presence of E6AP is shared by other HPV types causally associated with cervical cancer, we studied the potential interaction of the E6 oncoprotein derived from HPV-18. This revealed that a stable interaction with RhoA depends on the source of 18 E6 and that RhoA has only a slight inhibitory effect on 18 E6-E6AP-mediated ubiquitination in vitro (Supplementary Fig. 8). Thus, whereas 18 E6 has the potential to interact with RhoA, the interaction appears to be significantly less robust than the interaction between 16 E6 and RhoA. Furthermore, we could not observe an interaction of RhoA with the E6 protein derived from HPV-11 (Supplementary Fig. 8), which can also interact with E6AP^58^, but has not been associated with cervical cancer^2,70^. This differential interaction with RhoA potentially mirrors the affinity of the different E6 proteins for E6AP, with 16 E6 having the highest affinity and 11 E6 the lowest^71^. Thus, in future studies it will be of interest to identify the determinants of the E6 proteins that are required to drive E6AP into a RhoA-binding competent conformation.

In case of the interaction of the E6-E6AP complex with p53, E6AP stabilizes a p53-binding competent E6 conformation^50^, whereas for RhoA, it appears to be the other way around, i.e. E6 stabilizes a RhoA-binding competent conformation of E6AP. The latter raises the possibility that also in HPV-negative cells, mechanisms exist that enable E6AP to interact with RhoA and/or RhoA subfamily members with high affinity. For instance, proteins or other biomolecules may exist that act as E6 analogs inasmuch as they stabilize a RhoA-binding competent conformation of E6AP. Ephexin 5 acts as GEF for RhoA and was reported to be a substrate of E6AP^19^. As RhoA-GTP is selectively recognized by the E6-E6AP complex, we speculated that in addition to being a substrate of E6AP, Ephexin 5 may enable E6AP to interact with RhoA. However, we could not obtain any evidence for this hypothesis. Irrespectively, we favor the possibility that RhoA represents a substrate of E6AP rather than a regulator, as aberrant RhoA activation has been observed in an AS mouse model (i.e. in the absence of E6AP expression)^19,72^. Besides noncovalent interactions with proteins or other biomolecules, distinct posttranslational modifications of E6AP may promote its ability to interact with RhoA. Considering its critical role in different clinical pictures, it seems rather surprising how little is known about posttranslational modifications of E6AP in general and how these may affect E6AP function in particular. Thus, to understand how dysregulation of E6AP contributes to different human disorders, it will not only be important to characterize its substrate spectrum in different human cell types / tissues, but also to gain a comprehensive picture on the identity of posttranslational modifications that E6AP is subjected to and control its function.

## Methods

### Plasmids

cDNAs encoding RhoA, RhoB, RhoC, Rac1, and Cdc42 were kindly provided by C. Hauck (Dept. of Biology, University of Konstanz, Germany) and were inserted into pGEX-2TK and pcDNA3-HA for bacterial expression and in vitro translation/transfection experiments, respectively. cDNA encoding NHERF1 was a gift from Maria-Magdalena Georgescu (Addgene plasmid # 28291; http://n2t.net/addgene:28291; RRID: Addgene_28291)^73^ and was inserted into pcDNA3-HA for in vitro translation. For the generation of cDNAs encoding C-terminally truncated Rho variants or Rho variants harboring point mutations, the Q5 Site-Directed Mutagenesis Kit (New England Biolabs) was used. The construct encoding His-MBP-tagged HPV-16 E6 (His-MBP-E6) was kindly provided by G. Travé (IGBMC, Strasbourg, France). Bacterial expression constructs for UbcH7-His, His-E6AP, and GST-fusion proteins of HPV-16 E6 and Ulp1 as well as constructs for in vitro translation/transfection experiments encoding HA-tagged variants of E6AP, HPV-16 E6, OTUD5, DDX3X, and Ring1b I53S (catalytically inactive variant, substitution of I53 by S) were described previously^18,28,29,31,36,58,74,75^.

### Protein expression and purification

Proteins were expressed in *E. coli* BL21 gold (DE3) (GST-tagged and MBP-tagged proteins) or Rosetta 2 (DE3) (His-tagged proteins). Cultures were grown at 37 °C until an OD_600_ = 0.6–0.8 was reached. Protein expression was induced by adding 400 µM IPTG (final conc.). For GST-tagged proteins, cultures were grown for an additional 4 h at 37 °C; for MBP-tagged and His-tagged proteins, cultures were grown overnight at 20 °C. Upon centrifugation, pellets derived from 1 l bacterial culture were resuspended in 30 ml lysis buffer (1x PBS, 0.1% Triton X-100, 1 µg/ml aprotinin, 1 µg/ml leupeptin, 100 µM Pefabloc, 1 mM DTT), sonicated, centrifuged, and the supernatants used for purification.

Purification of His-E6AP and UBA1 was performed as described^31,76^. For GST-fusion proteins of HPV-16 E6 (GST-E6), Ulp1, and Rho proteins, the supernatant from 1 l bacterial culture was added to 600 µl GSH beads (1:1 mixture of Glutathione Sepharose 4B and Sepharose CL-4B; Cytiva) pre-equilibrated in lysis buffer. After incubation for 90 min at 4 °C, beads were washed 3x with 1 ml washing buffer (1x PBS, 0.1% Triton X-100, 1 mM DTT). The respective GST fusion protein was eluted with 5 × 300 µl elution buffer (50 mM Tris-HCl pH 8.0, 25 mM glutathione, 1 mM DTT). Eluates were analyzed by SDS-PAGE followed by Coomassie blue staining, and pooled eluates were dialyzed against 25 mM Tris-HCl pH 7.5, 50 mM NaCl, 1 mM DTT.

For generation of “tag-free” HPV-16 E6 and RhoA, HPV-16 E6 and RhoA were expressed as GST-SUMO fusion proteins. As described above, the supernatant derived from 1 l expression culture was incubated with 600 µl GSH beads pre-equilibrated in lysis buffer. After incubation for 90 min at 4 °C, beads were applied to a Poly-Prep Chromatography Column (Bio-Rad) and washed 3x with 10 ml washing buffer (1x PBS, 0.1% Triton X-100, 1 mM DTT). Beads were equilibrated with 10 ml SUMO protease buffer (50 mM Tris-HCl pH 8.0, 150 mM NaCl, 1 mM DTT, 5% (v/v) glycerol). For GST-Ulp1, the supernatant from 250 ml expression culture was bound to GSH beads and equilibrated in SUMO protease buffer. Then, the beads harboring the GST-SUMO fusion proteins were combined with the GST-Ulp1 beads in a ratio of 2:1 (v/v). Beads were incubated overnight at 4 °C in SUMO protease buffer. Upon application to a column, the flow-through was collected (elution 1). Beads were washed 3x with 1 ml of SUMO protease buffer, and the eluates analyzed by SDS-PAGE followed by Coomassie blue staining. Proteins were freshly prepared for each experiment.

For purification of His-MBP-E6 (during expression, 0.2% glucose were present in the culture media^77^) and UbcH7-His, the supernatant of a respective 1 l expression culture was filtered through a 0.45 μm syringe filter and applied to a Ni-NTA chromatography column (5 ml HisTrap HP, Cytiva) pre-equilibrated in buffer A (25 mM Tris-HCl pH 7.5, 50 mM NaCl, 1mM DTT). The column was washed with 2 column volumes (CV) of buffer A and 4 CV of 4% buffer B (25 mM Tris-HCl pH 7.5, 50 mM NaCl, 500 mM imidazole, 1mM DTT). Proteins were eluted with a gradient from 4% buffer B to 100% buffer B in 10 CV. Fractions were analyzed by SDS-PAGE followed by Commassie blue staining. Fractions containing His-MBP-E6 or UbcH7-His were pooled and dialyzed against 25 mM Tris-HCl pH 7.5, 50 mM NaCl, 1 mM DTT.

### In vitro ubiquitination

For ubiquitination of bacterially expressed GST-RhoA, 50 nM UBA1, 250 nM UbcH7-His, 250 nM His-E6AP, 20 µM ubiquitin (Sigma) and 45 µM GST-RhoA were incubated in ubiquitination buffer (25 mM Tris-HCl pH 7.5, 50 mM NaCl, 1 mM DTT, 2 mM ATP, 2 mM MgCl_2_) in presence or absence of 20 µM GST-E6 in a reaction volume of 20 µl for 90 min at 30 °C, unless stated otherwise. Reactions were stopped by the addition of 5x SDS loading buffer (312.5 mM Tris-HCl pH 6.8, 500 mM DTT, 10% SDS, 0.001% bromophenol blue), and the reactions were analyzed by electrophoresis on 12.5% SDS-PA gels followed by Coomassie blue staining.

For ubiquitination of in vitro translated ^35^S radiolabeled proteins, substrate proteins were translated in rabbit reticulocyte lysate or wheat germ extract (T7-coupled TNT translation systems; Promega) as indicated. In vitro ubiquitination assays were performed in 20 µl volumes containing 1 µl of in vitro translated radiolabeled protein, 50 nM UBA1, 250 nM UbcH7-His, 250 nM His-E6AP, 50 µM ubiquitin (Sigma) in ubiquitination buffer in presence or absence of 20 µM GST-E6 and 30–100 µM GST-RhoA for 90 min at 30 °C, unless stated otherwise. Note that in the experiments to Fig. 2 and Supplementary Fig. 3, a hydrophobic patch mutant of ubiquitin (Ub-LIA) instead of commercial wild-type ubiquitin was employed. In contrast to wild-type ubiquitin, Ub-LIA is efficiently used by the E6-E6AP complex, but less efficiently by E6AP alone^28^. Thus, Ub-LIA is particularly well suited to monitor the activity of the E6-E6AP complex, but similar results were obtained with wild-type ubiquitin. Ubiquitination reactions were stopped by the addition of 5x SDS loading buffer, and whole reaction mixtures were analyzed by electrophoresis on 10–15% SDS-PA gels depending on the substrate. Gels were fixed for 10 min in fixing solution (10% acetic acid, 40% methanol), incubated in Lightning Autoradiography Enhancer Solution (PerkinElmer) for 20 min, vacuum-dried at 80 °C, and finally subjected to a BAS MS-2040 imaging plate (Fujifilm). Signals were read out by a FLA-5000 scanner (Fujifilm).

### In vitro coprecipitation assays

For in vitro coprecipitation assays, GSH beads (1:1 mixture of Glutathione Sepharose 4B and Sepharose CL-4B) were equilibrated in PBS-T lysis buffer (1x PBS, 0.1% Triton X-100, 1 µg/ml aprotinin, 1 µg/ml leupeptin, 100 µM Pefabloc, 1 mM DTT) and incubated with cleared bacterial lysate containing the GST fusion protein of interest (GST-POI) at 4 °C in a ratio (v/v) of beads to lysate of 1:1500. After 90 min, beads were washed 3x with 1 ml of PBS-T lysis buffer and once with 1 ml pulldown buffer (40 mM Tris-HCl pH 7.6, 90 mM NaCl, 0.2% (v/v) IGEPAL, 1 mM DTT). Coprecipitation assays were performed in a total volume of 200 µl in pulldown buffer with 20 µl beads (harboring the respective GST-POI), 10 µl of in vitro translated radiolabeled protein, and additional proteins of interest indicated in the respective figure legends. Binding mixtures were incubated for 90 min at 4 °C, and unbound proteins were removed by washing 3x with 1 ml TNN buffer (100 mM Tris-HCl pH 8.0, 100 mM NaCl, 1% (v/v) IGEPAL, 1 mM DTT). Washed beads were boiled in 15 µl 2x SDS loading buffer (125 mM Tris-HCl pH 6.8, 200 mM DTT, 4% SDS, 0.001% bromophenol blue) and subjected to SDS-PAGE. Gels were first analyzed by Coomassie blue staining to determine the input levels of the GST-POI and subsequently analyzed by fluorography to determine the levels of the radiolabeled protein bound to the GST-POI.

### In vitro interaction analysis by spectral shift measurement

Spectral shift measurements were performed on a Monolith X instrument (Nanotemper) using Monolith Premium Capillaries. For the response evaluation, the spectral shift 670 nm/650 nm ratio was plotted against the ligand concentration using the MO.Control Software (Nanotemper), and binding affinities were determined with the Kd model provided by the software. The measurements were carried out at 25 °C in 25 mM Tris-HCl pH 7.5, 50 mM NaCl, 0.01% Tween 20. The target (GST-tagged RhoA G14V) was labelled with the Protein Labeling Kit RED-NHS 2nd Generation (Nanotemper, MO-L011) according to the manufacturer’s instructions. During the measurement, a constant target concentration (0.05 µM) was supplemented with a dilution series (16 samples ranging from 0 up to the indicated maximum concentration, see Table 2) of the respective ligand (4.9 µM His-E6AP or 8.4 µM GST-16 E6). Based on the binding observed between E6AP and GST-RhoA G14V but not between E6 and GST-RhoA G14V, the ternary binding affinity was measured with GST-RhoA G14V (0.05 µM), E6AP (2.45 µM) defined as the primary interactor and E6 as ligand (serial dilution up to 8.4 µM).

### Transient transfection

H1299 cells (ATCC, #CRL-5803) were cultivated at 37 °C, 95% humidity and 5% CO2 in DMEM (Gibco) containing 10% (v/v) FCS. For transient transfection experiments, cells were seeded in 6-well tissue culture plates (Sarstedt) in a total volume of 2 ml. At 70% confluency, cells were transfected using Lipofectamine 2000 (Invitrogen) according to the manufacturer’s instructions.

Transfections were performed with expression constructs encoding HA-tagged proteins as indicated. In addition, a β-galactosidase expression construct was cotransfected to determine relative transfection efficiencies, and total DNA amounts in all transfections were equalized by addition of empty vector (pcDNA3). 24 h after transfection, cells of one well were scraped (Cell lifter, Corning Inc.) with 700 µl ice-cold PBS and harvested by centrifugation. Cell pellets were lysed in 80 µl TNN lysis buffer (100 mM Tris-HCl pH 8.0, 100 mM NaCl, 1% (v/v) IGEPAL, 1 µg/ml aprotinin, 1 µg/ml leupeptin, 100 µM Pefabloc, 1 mM DTT) for 30 min on ice. The lysate was cleared by centrifugation, and volumes of lysates used for SDS-PAGE and western blotting analysis were adjusted based on transfection efficiencies, as determined by measuring β-galactosidase activity in duplicates. Levels of HA-tagged proteins were monitored by the mouse monoclonal HA.11 antibody (Covance), and p53 was detected with the mouse monoclonal DO-I antibody (Calbiochem).

### XL-MS with DSS

For crosslinking with disuccinimidyl suberate (DSS), purified His-E6AP, GST-E6, and untagged RhoA G14V were incubated in different combinations (molar ratio of E6AP : E6 : RhoA 1 : 1.5 : 2.5; equalized across samples to a total protein concentration of 0.3 µg/µl) in crosslinking buffer (25 mM Tris-HCl pH 7.5, 50 mM NaCl, 1 mM DTT) for 30 min on ice. Then, the mixtures were crosslinked by addition of 1 mM H12/D12 DSS (in DMSO; Creative Molecules) and gentle shaking for 30 min at 37 °C. After quenching by addition of ammonium bicarbonate to a final concentration of 50 mM, samples were dried and resuspended in 8 M urea. Proteins were reduced with 2.5 mM TCEP for 30 min at 37 °C, alkylated with 5 mM iodoacetamide for 30 min at 23 °C in the dark, and digested with trypsin at enzyme:substrate (E:S) ratio of 1:40 for 18 h at 37 °C. The resulting peptides were desalted by C18 cartridges (SepPak, Waters), crosslinked peptides were enriched by size exclusion chromatography (Superdex 3.2/100, GL science), and fractions 1.0-1.5 ml were retained and dried for subsequent measurements. Peptides were resuspended in MS buffer (5% acetonitrile, 0.1% formic acid) and separated across a 48 min gradient on a 15 cm Acclaim™ PepMap™ C18 column (P/N164943) using an EASY-nLC™ 1200 system connected to an Orbitrap Fusion Tribrid mass spectrometer (both Thermo Scientific). MS measurement was performed in data-dependent acquisition mode with a cycle time of 3 s. The full scan was done in the Orbitrap with a resolution of 120 K, a scan range of 400–1650 m/z, AGC Target 2.0e5 and injection time of 50 ms. Monoisotopic precursor selection and dynamic exclusion for 60 s was used for precursor selection. Only precursor charge states of 3–8 were selected for fragmentation by CID using 35% activation energy. MS2 was carried out in the Ion Trap in rapid scan rate mode, AGC target 1.0e4 and dynamic injection time.

Raw data was converted to centroided mzxml files and searched for crosslinked peptides using xQuest^78^ in ion-tag mode with a precursor mass tolerance of 10 ppm. For matching of fragment ions, tolerances of 0.2 Da for common ions and 0.3 Da for crosslink ions were applied. Crosslinked samples were prepared in biological triplicates (i.e. separately expressed and purified batches of proteins) for all investigated samples, and each of these was measured in technical duplicates. Crosslinks were only considered during interaction analysis, if they were identified in at least 2 of 3 biological replicates with deltaS < 0.95, an Id score ≥ 25, and FDR < 10%.

### XL-MS with Sulfo-SDA

Crosslinking of His-E6AP, GST-E6 and RhoA G14V with sulfo-succinimidyl 4,4’-azipentanoate (SDA) was performed as described for DSS except for the following details. Total protein concentration across samples was equalized to 0.2 µg/µl. Crosslinking with 1 mM sulfo-SDA was performed at 37 °C for 30 min in the dark, then the reaction was quenched with 50 mM ammonium bicarbonate, and the second part of the reaction was performed in a Bio-Link BLX for 15 min at 365 nm and maximum power on ice. After size exclusion chromatography, fractions 1.0-1.6 ml were retained and dried.

MS measurement was performed in data-dependent mode on an Orbitrap Tribrid Fusion mass spectrometer with a maximum cycle time of 3 s. Full scans were acquired in the Orbitrap at 120 K, a scan range of 375–1650 m/z, AGC target 2e5 and injection time of 50 ms. Selected precursors were fragmented by 30% HCD. MS2 scans were acquired in the Orbitrap at 30 K, AGC target 5e4 and a dynamic injection time.

Data was converted to mgf files and searched for crosslinks using MeroX^79,80^. Carbamidomethylation was set as static modifications for cysteines, oxidation as variable modification for methionines, and missed cleavages was set to 3. SDA was introduced as crosslinker with a specificity for K, S, T, and Y on the NHS ester site, and all proteogenic amino acids as specificity on the second site. Dead-end and intrapeptide crosslinks were enabled and consecutive peptides as crosslinks were ignored. Precursor precision was set to 5 ppm and fragment precision to 10 ppm and data was analyzed in quadratic mode. Crosslinks were only considered for interaction analysis with FDR ≤ 5% and score ≥ 70.

### LiP-MS

Limited proteolysis coupled to mass spectrometry (LiP-MS) experiments were performed essentially as described^81^. 20 µg of protein or protein mixtures (molar ratio of E6AP:E6:RhoA of 1:1.5:2.5) at a total concentration of 0.2 µg/µl were mixed and incubated for 30 min on ice, then split into Proteinase K (PK) and control conditions and equilibrated to 25 °C prior to LiP. The experiment was performed in biological quadruplicates. For LiP, 5 µl of 8 µg/ml Proteinase K (from *Engydontium album*, E:S ratio of 1:250) or 5 µl MilliQ for the control samples were added simultaneously, mixed by pipetting and samples were incubated at 25 °C for 5 min before the enzymatic digestion was stopped by heating to 99 °C for 5 min. After cooling to room temperature, sodium deoxycholate (SOD) was added to a final concentration of 5% (w/v). Proteins were reduced with 2.5 mM TCEP for 30 min at 37 °C and alkylated with 5 mM iodoacetamide for 30 min at 23 °C in the dark. After dilution of SOD to 1%, Lys-C/trypsin mix (Promega V5071, Sequencing grade) was added at an E:S ratio of 1:50 and incubated for 18 h at 37 °C. The enzymatic digestion was stopped, and SOD precipitated by addition of 98% formic acid (FA) to a final concentration of 2%. The SOD precipitate was removed by centrifugation at 16,000 g for 5 min and remaining detergent removed by Ultrafree^®^-MC spin columns (Millipore, #UFC30HV) before peptides were desalted with C18 Spin tips (Pierce, #84850) and dried.

For MS analysis, dried peptides were resuspended in 2% ACN, 0.1% FA. For retention time normalization, iRT peptides (Biognosys) were added at a ratio of 1:40. Approximately 500 ng peptides were loaded onto a 15 cm Acclaim™ PepMap™ C18 column (P/N 164943, Thermo Scientific) connected to an EASY-nLC™ 1200 system and separated across a 75 min gradient. Mass spectra were acquired on an Orbitrap Tribrid Fusion mass spectrometer (Thermo Scientific) in data-independent mode using 18 variable windows that were adapted to the m/z density distribution of ions in the samples^82^. Precursor mass spectra were acquired in the Orbitrap at 120 K over a mass range of 300–1500 m/z using 50 ms maximum injection time (max IT) and an automatic gain control (AGC) target of 5e4. Fragment mass spectra were acquired in the Orbitrap at 30 K with an AGC target of 4e6, while max IT was set to auto. Precursors were fragmented with stepped HCD at 30 ± 4%. Raw data was analyzed separately for PK and control samples with Spectronaut version 18.6.231227.55695 in directDIA+ (Deep) mode. BGS factory settings were used with the exception that digest type was set to unspecific for the PK samples, minimum peptide length was set to 6, while imputation was disabled in the DIA quantification tab. Data was exported as MSstats reports and post-analysis processing including correction for protein abundance changes and imputation using the MSstatsLiP package as recommended^81^. Processed LiP data were exported, and results were filtered for statistically significant peptides (adjusted p-value < 0.05 and log2ratio >|1|) and visualized using python packages pandas (version 1.4.4, https://pandas.pydata.org/), numpy (version 1.21.5, https://www.numpy.org) and plotly (plotly version 5.9.0, https://plotly.com/python/) running on python (version 3.9.13). The sequence plot code was adapted from Schessner et al., 2022^83^.

## Supplementary Information

Below is the link to the electronic supplementary material.

Supplementary Material 1

## Data Availability

The mass spectrometry data (raw files, xQuest, MeroX, and Spectronaut search output files) have been deposited to the ProteomeXchangeConsortium via the PRIDE repository^84^. The crosslink dataset has the identifier PXD069396 (Token: CQvaMWI54QYP; alternatively: Username: reviewer_pxd069396@ebi.ac.uk, Password: uHIIF7FXKcLE), the LiP-MS dataset has the identifier PXD069419 (Token: Zu5oft5ai9k9; alternatively: Username: reviewer_pxd069419@ebi.ac.uk, Password: JTl6Du4DFarw), and the gel-based MS dataset has the identifier PXD069463 (Token: vaz2SCjxE6ds; alternatively: Username: reviewer_pxd069463@ebi.ac.uk, Password: hXUA89pMyA17). Other data generated in this study are provided in this article and its supplementary information file.
