# Supplementary material for: Members of the RhoA subfamily of GTPases suppress the stimulatory effect of the HPV E6 oncoprotein on the ubiquitin ligase E6AP
Source: Sci Rep. 2026 Aug 25;16:26683. doi: 10.1038/s41598-026-67837-8 (PMC13507084; doi:10.1038/s41598-026-67837-8)
Supplement: Supplementary file 1 [file 41598_2026_67837_MOESM1_ESM.pdf]

Members of the RhoA subfamily of GTPases suppress the stimulatory effect of the HPV E6 oncoprotein on the ubiquitin ligase E6AP

Daniela Eichbichler, Jasmin Jansen, Nadja Eulich, Florian Stengel, Martin Scheffner

Supplementary Figures 1-8

Full-length gels of Figures 1-4, 6, and of Supplementary Figures 1-4, 7, 8

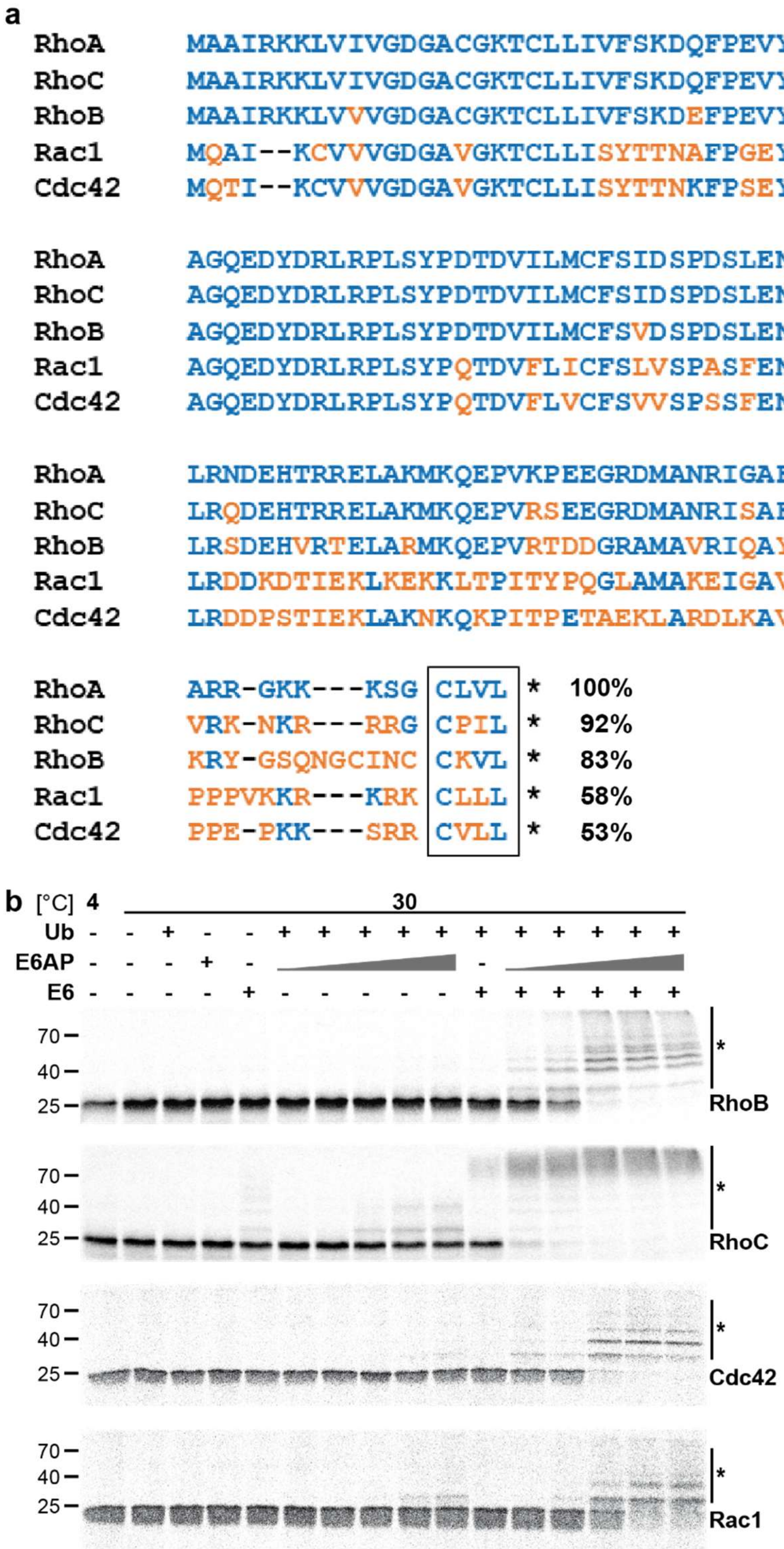

Supplementary Fig. 1. a, Sequence alignment of RhoA, RhoC, RhoB, Rac1, and Cdc42. Amino acids identical to the RhoA sequence are indicated in blue, amino acids different to the RhoA sequence are in orange. The asterisk represents the stop codon. The C-terminal CAAX motif is boxed. Amino acid sequence identity of the different proteins to RhoA is indicated. **b,** In vitro ubiquitination of RhoB, RhoC, Cdc42, and Rac1. In vitro translated, radiolabeled RhoB, RhoC, Cdc42, or Rac1 were incubated with increasing concentrations of recombinant E6AP in the absence or presence of the E6 oncoprotein under standard ubiquitination conditions (Methods) as indicated. After 90 min at 30 °C, whole reaction mixtures were analyzed by SDS-PAGE followed by fluorography. Running positions of molecular mass markers (kDa), unmodified RhoB/RhoC/Cdc42/Rac1, and ubiquitinated forms of RhoB/RhoC/Cdc42/Rac1 (asterisk) are indicated.

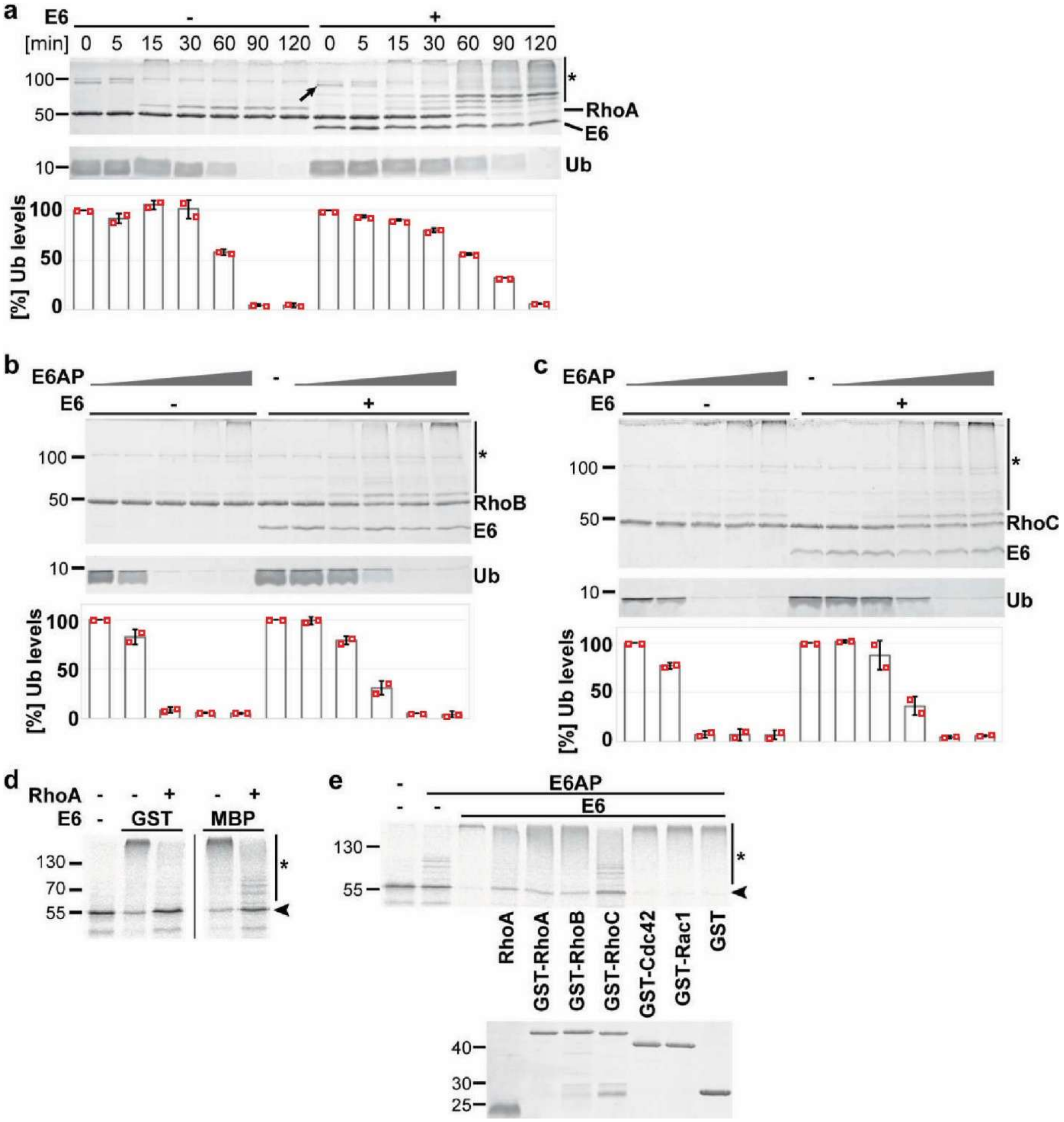

Supplementary Fig. 2. a-c, Bacterially expressed RhoA/RhoB/RhoC are ubiquitinated by E6-E6AP. **a,** GST-RhoA was incubated with recombinant E6AP in the presence or absence of E6 at 30 °C as indicated under standard ubiquitination conditions (see Methods). Reactions were stopped at the times indicated and analyzed by SDS-PAGE followed by Coomassie blue staining (upper panels). **b, c,** GST-RhoB or GST-RhoC were incubated with increasing concentrations of recombinant E6AP in the presence or absence of E6 as indicated under standard ubiquitination conditions (see Methods). After 90 min at 30 °C, reactions were stopped and analyzed by SDS-PAGE followed by Coomassie blue staining (upper panels). **a-c,** Relative levels of free ubiquitin (Ub) were determined by densitometry, with levels of ubiquitin at t = 0 min set to 100% (lower panels). Error bars indicate SD of the mean of two independent experiments. Running positions of molecular mass markers (kDa), unmodified forms of RhoA/RhoB/RhoC, E6, free ubiquitin (Ub), and E6AP (arrow) are indicated. Ubiquitinated forms of RhoA/RhoB/RhoC and E6AP are marked with an asterisk (note that poly-ubiquitinated forms of E6AP and RhoA cannot be distinguished).

d, RhoA blocks E6-E6AP-mediated ubiquitination irrespective of whether GST-tagged E6 or MBP-tagged E6 is used. In vitro translated, radiolabeled p53 was incubated with recombinant E6AP and with either GST-tagged E6 or MBP-tagged E6 in the absence or presence of GST-tagged RhoA as indicated under standard ubiquitination conditions (Methods). After 90 min at 30 °C, reactions were

stopped and analyzed by SDS-PAGE followed by fluorography. Running positions of molecular mass markers (kDa), unmodified p53 (arrowhead), and ubiquitinated forms of p53 (asterisk) are indicated. **e,** Rac1 and Cdc42 do not inhibit E6-E6AP-mediated ubiquitination of p53. In vitro translated, radiolabeled p53 was incubated recombinant E6AP and GST-E6 in the absence or presence of untagged RhoA and GST fusion proteins of RhoA, RhoB, RhoC, Cdc42, and Rac1 as indicated under standard ubiquitination conditions (Methods). After 90 min at 30 °C, reactions were stopped and analyzed by SDS-PAGE followed by fluorography. Running positions of molecular mass markers (kDa), unmodified p53 (arrowhead), and ubiquitinated forms of p53 (asterisk) are indicated. Lower panel, input levels of the indicated proteins analyzed by SDS-PAGE followed by Coomassie blue staining.

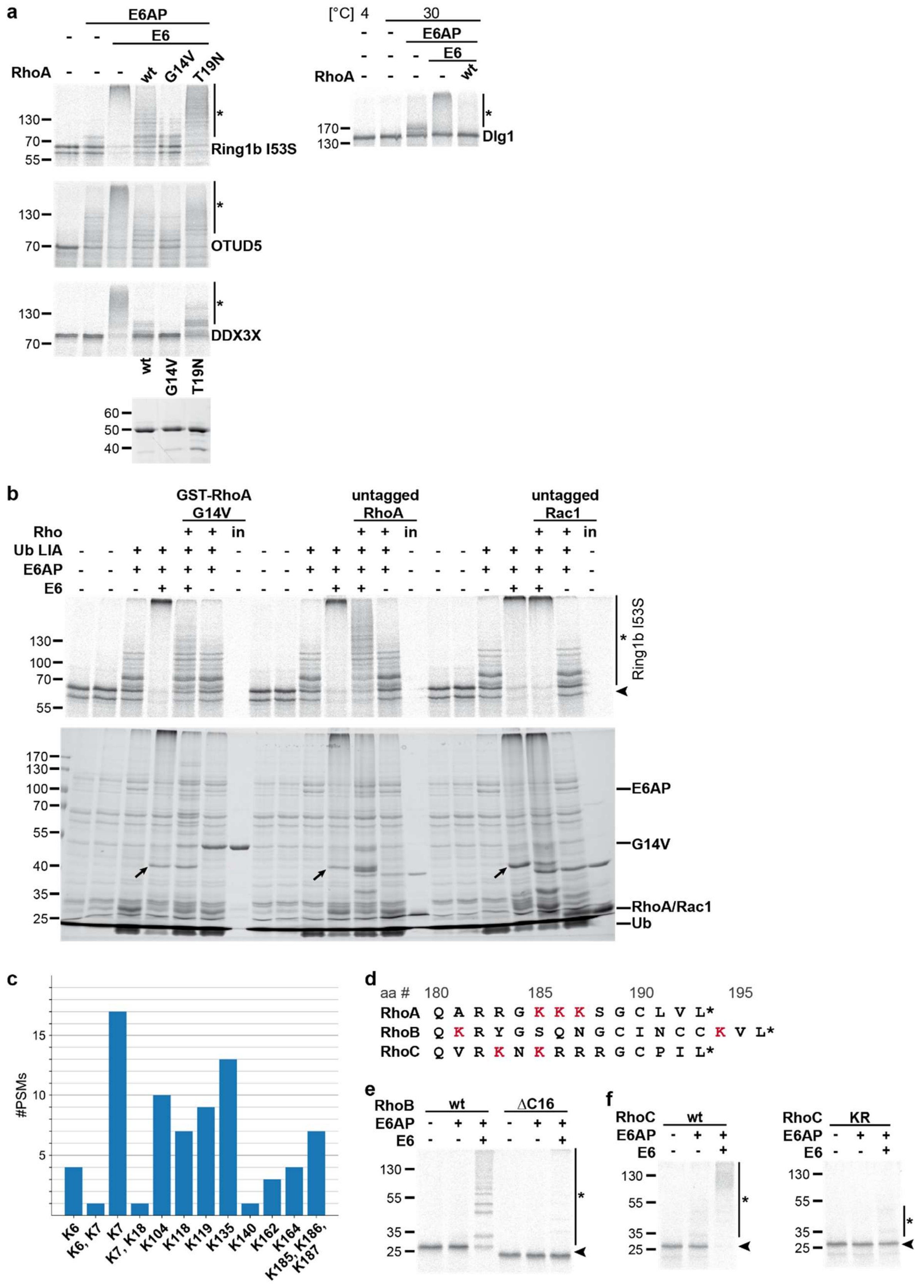

Supplementary Fig. 3. RhoA-G14V mimicking the active state of RhoA is most efficient in inhibiting the E3 activity of the E6-E6AP complex. **a,** In vitro translated, radiolabeled Ring1b I53S (catalytically inactive mutant of Ring1b), OTUD5, DDX3X, or Dlg1 were incubated with recombinant E6AP in the absence or presence of E6 and GST fusion proteins of wild-type (wt) RhoA and RhoA variants mimicking the active (G14V) and inactive (T19N) state as indicated under standard ubiquitination conditions (Methods). After 90 min at 30 °C, whole reaction mixtures were analyzed by SDS-PAGE followed by fluorography (upper panel). Running positions of molecular mass markers (kDa), unmodified proteins (Ring1b I53S, OTUD5, DDX3X), and the respective ubiquitinated forms (asterisk)

are indicated. Lower panel, input levels of the indicated proteins analyzed by SDS-PAGE followed by Coomassie blue staining. **b,** In vitro translated, radiolabeled Ring1b I53S was incubated with recombinant E6AP in the absence or presence of E6, a ubiquitin variant (Ub LIA)[1], and GST-tagged RhoA G14V, untagged RhoA or untagged Rac1 as indicated under standard ubiquitination conditions (Methods). After 90 min at 30 °C, whole reaction mixtures were analyzed by SDS-PAGE followed by Coomassie blue staining (lower panel) to monitor levels of E6AP, GST-E6, GST-RhoA G14V, RhoA, and Rac1, and subsequently by fluorography (upper panel) to detect radiolabeled Ring1b I53S. Note that RhoA and RhoA G14V blocked E6-E6AP-mediated ubiquitination of Ring1b I53S, but not E6AP-mediated ubiquitination of Ring1b I53S in the absence of E6. Running positions of molecular mass markers (kDa), unmodified Ring1b I53S (arrowhead), ubiquitinated forms of Ring1b I53S (asterisk), Ub LIA (Ub), GST-E6 (arrow), E6AP, GST-RhoA G14V, RhoA, and Rac1 are indicated. Input (in), 10% of RhoA G14V, RhoA, and Rac1 used in the ubiquitination reaction. **c,** Mass spectrometric determination of the lysine residues of RhoA that serve as attachment sites of ubiquitin. GST-tagged RhoA was incubated with recombinant E6AP and E6 under standard ubiquitination conditions (Methods). After 90 min at 30 °C, whole reaction mixtures were subjected to SDS-PAGE followed by Coomassie blue staining. Bands corresponding to ubiquitinated forms of RhoA were excised, processed by in-gel digestion[2], and analyzed by LC-MS/MS in DDA mode. **d,** Amino acid sequence of the C-terminal region of RhoA, RhoB, and RhoC, with lysine residues highlighted in red. Numbering is according to the RhoA sequence. Asterisk, denotes the respective stop codon. **e,** The C-terminal 16 amino acid residues of RhoB are required for ubiquitination. In vitro translated, radiolabeled wild-type (wt) RhoB or a RhoB mutant lacking the C-terminal 16 amino acids (ΔC16) were incubated with recombinant E6AP in the absence or presence of E6 as indicated under standard ubiquitination conditions (Methods). After 90 min at 30 °C, whole reaction mixtures were analyzed by SDS-PAGE followed by fluorography. Running positions of molecular mass markers (kDa), unmodified RhoB variants (arrowhead), and ubiquitinated forms thereof (asterisk) are indicated. **f,** K183 and K185 of RhoC serve as main attachment sites for ubiquitin. In vitro translated, radiolabeled wild-type (wt) RhoC and RhoC KR (substitution of K183 and K185 by R) were incubated with recombinant E6AP in the absence or presence of E6 as indicated under standard ubiquitination conditions (Methods). After 90 min at 30 °C, whole reaction mixtures were analyzed by SDS-PAGE followed by fluorography. Running positions of molecular mass markers (kDa), unmodified wt RhoC and RhoC KR (arrowhead) and respectively ubiquitinated forms (asterisk) are indicated.

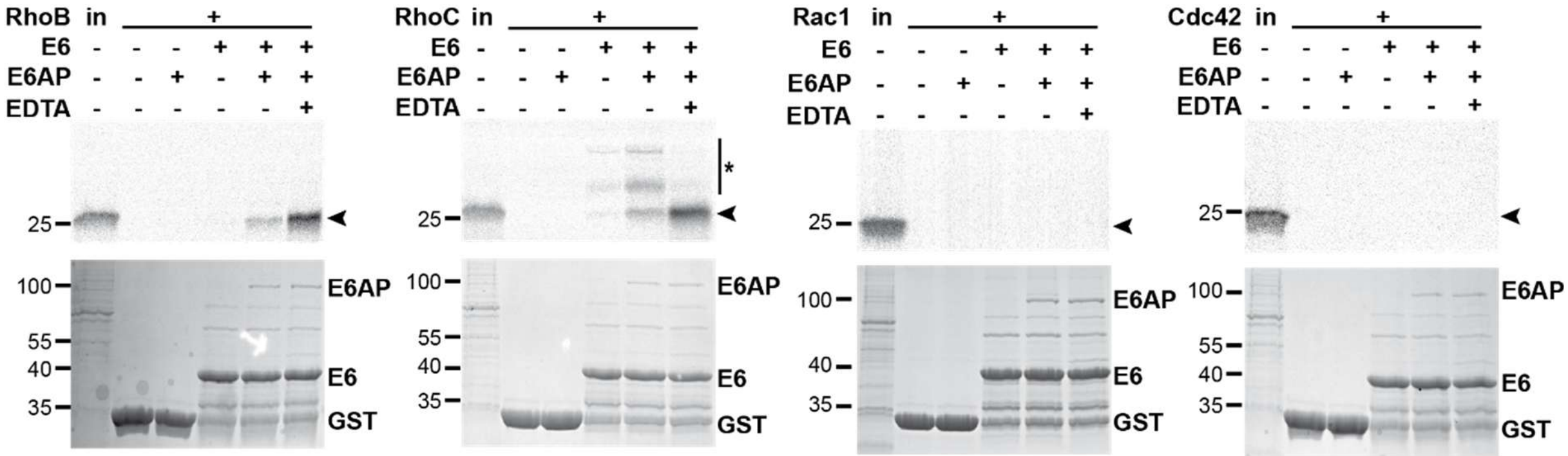

Supplementary Fig. 4. Members of the RhoA subfamily, but not Cdc42 and Rac1 are able to form a ternary complex with E6 and E6AP. GST-tagged E6 bound to GSH beads was incubated with in vitro translated, radiolabeled RhoB, RhoC, Rac1, or Cdc42 in the absence or presence of recombinant E6AP as indicated. As control, GSH beads decorated with GST alone were used. After 90 min at 4 °C, beads were washed and bound proteins analyzed by SDS-PAGE followed by Coomassie blue staining (lower panel) to monitor levels of GST-E6 and E6AP and subsequently by fluorography (upper panel) to detect radiolabeled RhoB/RhoC/Cdc42/Rac1. Running positions of molecular mass markers (kDa), unmodified RhoB/RhoC/Cdc42/Rac1 (arrowhead), ubiquitinated forms of RhoC (asterisk), GST-E6, GST, and E6AP are indicated. Input (in), 10% of in vitro translated RhoB/RhoC/Cdc42/Rac1 used in the binding reaction.

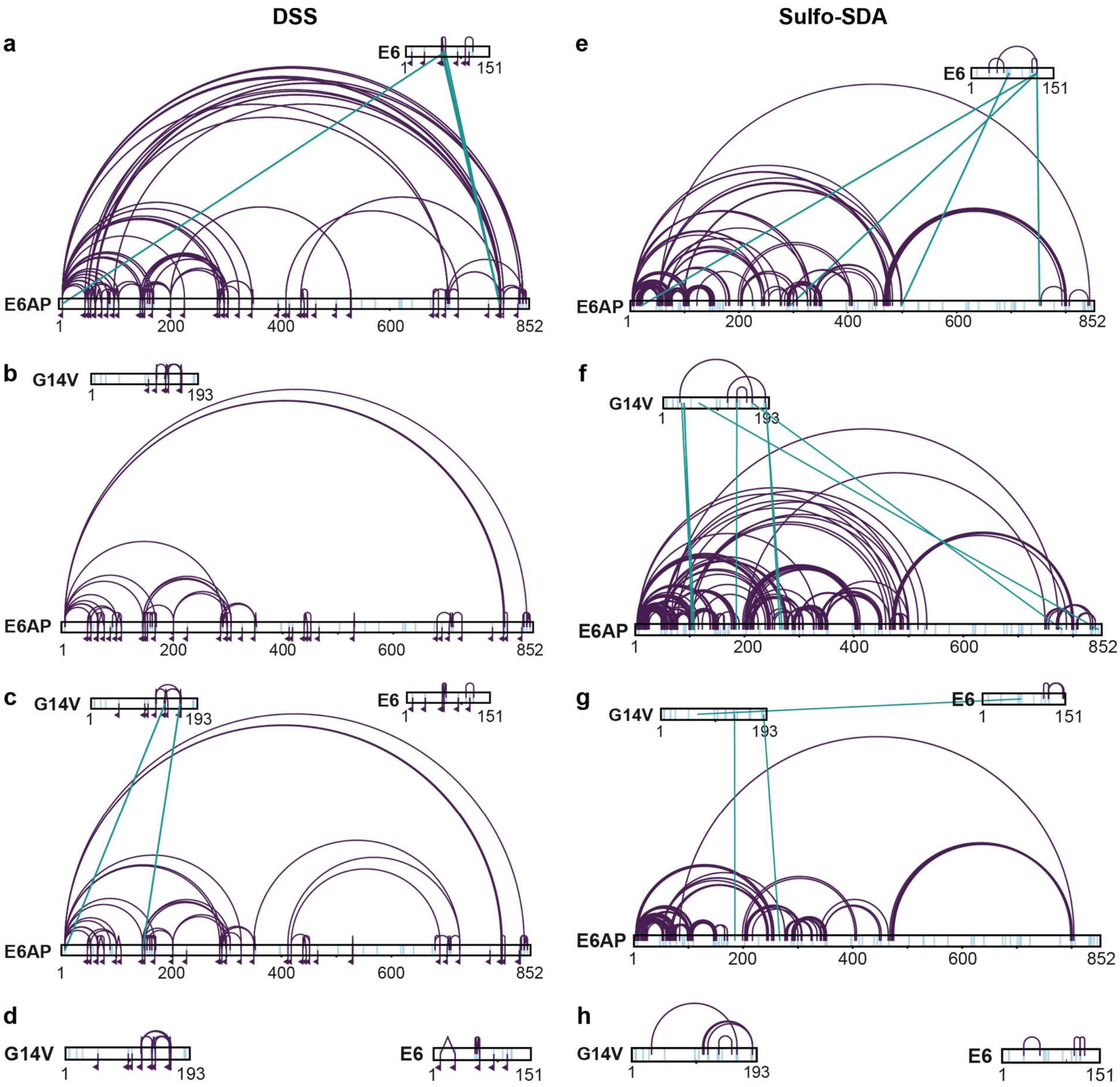

Supplementary Fig. 5. Comparison of E6AP inter- (green) and intra-protein crosslink (purple) patterns formed with two different crosslinkers, DSS or Sulfo-SDA, in the presence of **a,e** E6, **b,f** RhoA-G14V, **c,g** E6 and RhoA-G14V. **d,h,** crosslink pattern of E6 and RhoA-G14V in the absence of E6AP. Purple flags indicate mono-links (hydrolyzed crosslinker); positions of lysine residues are indicated in light blue. For details, see Methods.

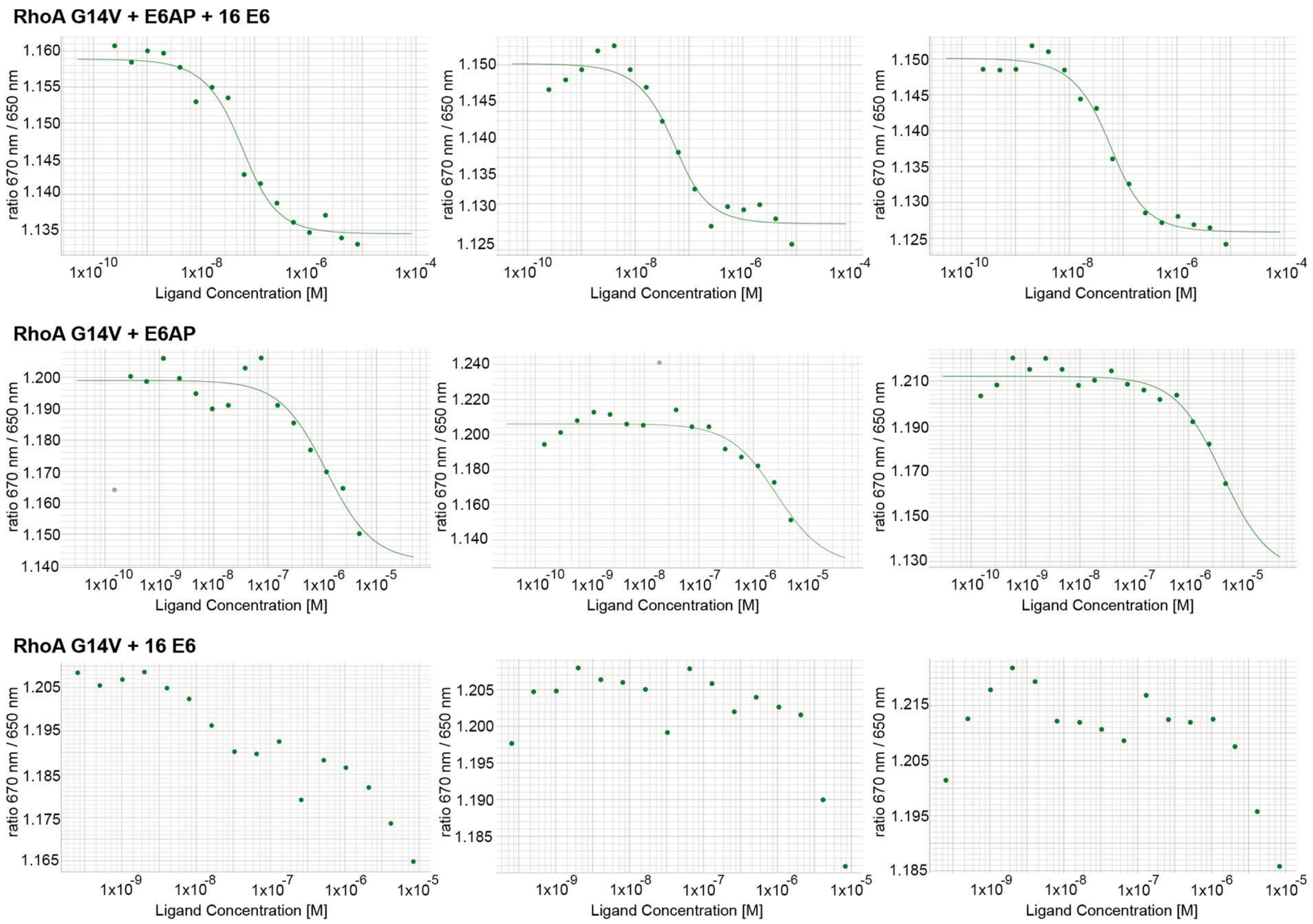

Supplementary Fig. 6. Graphs of the spectral shift analysis of the interaction of fluorescently labeled GST-RhoA G14V with E6 or E6AP or both that were used for Kd determination (Table 2). Spectral shift (670 nm/650 nm ratio) was blotted against the ligand concentration (in M). For further information, see Table 2.

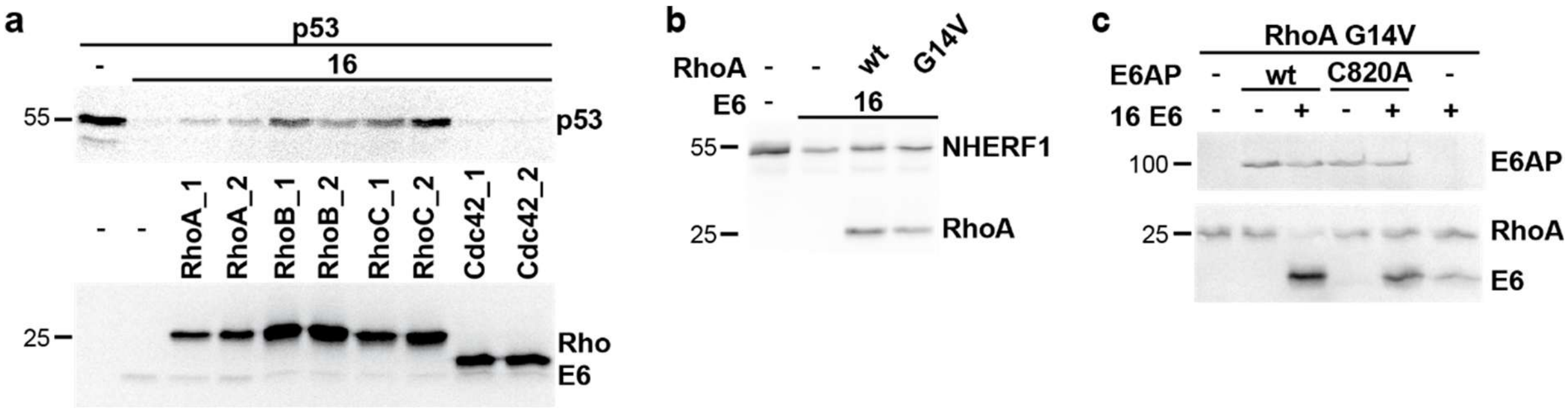

Supplementary Fig. 7. a, H1299 cells were transfected with expression constructs encoding HA-tagged forms of p53 and E6 and two different preprations of expression constructs encoding Ha-tagged forms of RhoA, RhoB, RhoC, and Cdc42 as indicated. 24 h post-transfection, cell lysates were prepared, and transfection efficiency adjusted aliquots (Methods) were subjected to SDS-PAGE followed by western blot analysis using the p53-specific antibody DO-1 (upper panel) or an anti-HA antibody (lower panel). Running positions of molecular mass markers (kDa), p53, E6, and RhoA/B/C and Cdc42 (Rho) are indicated. **b,** H1299 cells were transfected with expression constructs encoding HA-tagged forms of NHERF1, E6, RhoA (wt) and RhoA G14V as indicated. 24 h post-transfection, cell lysates were prepared, and transfection efficiency adjusted aliquots (Methods) were subjected to SDS-PAGE followed by western blot analysis using an anti-HA antibody. Running positions of molecular mass markers (kDa) are indicated. Note that under the conditions used, levels of HA-tagged E6 were too low to be detected by western blot. **c,** E6-mediated degradation of RhoA depends on the catalytic activity of E6AP. H1299-K3 cells (stable knockdown of endogenous E6AP expression[3]) were transfected with expression constructs encoding HA-tagged forms of E6AP, a catalytically inactive variant of E6AP (C820A; substitution of the catalytic cysteine residue by alanine), E6, and RhoA G14V as indicated. 24 h post-transfection, cell lysates were prepared, and transfection efficiency adjusted aliquots (Methods) were subjected to SDS-PAGE followed by western blot analysis using an anti-HA antibody. Running positions of E6AP, E6, and RhoA G14V are indicated. Note that as reported[4], coexpression of E6AP results in increased levels of HA-tagged E6.

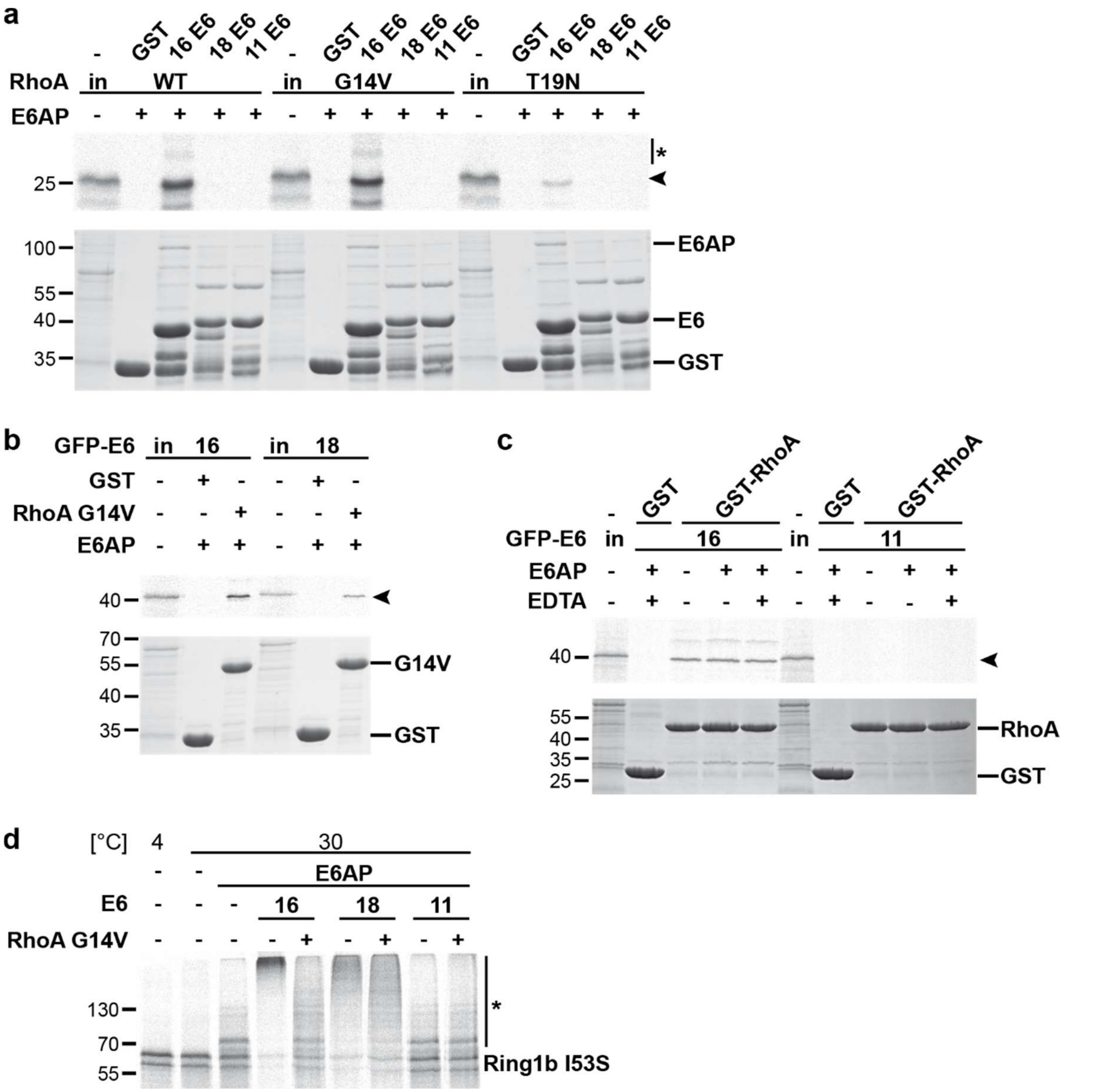

Supplementary Fig. 8. a, GST-tagged E6 (E6) proteins derived from different HPV types (16, 18, 11) bound to glutathione sepharose (GSH) beads were incubated with in vitro translated, radiolabeled RhoA (wt), Rho G14V, or RhoA T19N in the presence of recombinant E6AP and 20 mM EDTA as indicated. As control, GSH beads decorated with GST alone were used. After 90 min at 4 °C, beads were washed and bound proteins were analyzed by SDS-PAGE followed by Coomassie blue staining (lower panel) to monitor levels of GST, the GST-E6 proteins, and E6AP, and subsequently by fluorography (upper panel) to detect radiolabeled RhoA variants. Running positions of molecular mass markers (kDa), RhoA (arrowhead), GST-E6, GST, and E6AP are indicated. Input (in), 10% of in vitro translated RhoA used in the binding reaction. **b, c,** GST or GST-tagged RhoA bound to GSH beads (were incubated with in vitro translated, radiolabeled GFP fusion proteins of 16 E6 and 18 E6 (b) or of 16 E6 and 11 E6 (c) in the absence and presence of recombinant E6AP and EDTA as indicated. After 90 min at 4 °C, beads were washed, and bound proteins were analyzed by SDS-PAGE followed by Coomassie blue staining (lower panel) to monitor levels of GST-RhoA and GST, and subsequently by fluorography (upper panel) to detect radiolabeled E6 proteins. Running positions of molecular mass markers (kDa), GST-RhoA, GST, and the E6 proteins are indicated. Input (in), 10% of in vitro translated E6 proteins used in the binding reaction. **d,** In vitro translated, radiolabeled Ring1b I53S (catalytically inactive mutant of Ring1b) was incubated with a ubiquitin variant (Ub LIA)[1] and recombinant E6AP in the absence or presence of GST E6 fusion proteins derived from the HPV types indicated (16, 18, 11) and a GST fusion protein of RhoA G14V as indicated under standard ubiquitination conditions (Methods). After 90 min at 30 °C, whole reaction mixtures were analyzed by

SDS-PAGE followed by fluorography. Whereas RhoA has a strong inhibitory effect on 16 E6-stimulated ubiquitination of Ring1b I53S, it has a rather mild effect on 18 E6-stimulated ubiquitination of Ring1b I53S (as judged by the amount of highly ubiquitinated forms of Ring1b I53S migrating at the top of the gel). Running positions of molecular mass markers (kDa), unmodified Ring1b I53S (arrowhead), ubiquitinated forms of Ring1b I53S (asterisk) are indicated.

References

1. Mortensen, F. et al. Role of ubiquitin and the HPV E6 oncoprotein in E6AP-mediated ubiquitination. *Proc. Natl. Acad. Sci. U S A* **112**, 9872-9877 (2015).
2. Shevchenko, A., Tomas, H., Havlis, J., Olsen, J.V. & Mann, M. In-gel digestion for mass spectrometric characterization of proteins and proteomes. *Nat. Protoc.* **1**, 2856-2860 (2006).
3. Kuballa, P., Matentzoglu, K. & Scheffner, M. The role of the ubiquitin ligase E6-AP in human papillomavirus E6-mediated degradation of PDZ domain-containing proteins. *J. Biol. Chem.* **282**, 65-71 (2007).
4. Tomaic, V., Pim, D. & Banks, L. The stability of the human papillomavirus E6 oncoprotein is E6AP dependent. *Virology* **393**, 7-10 (2009).

Full-length gels

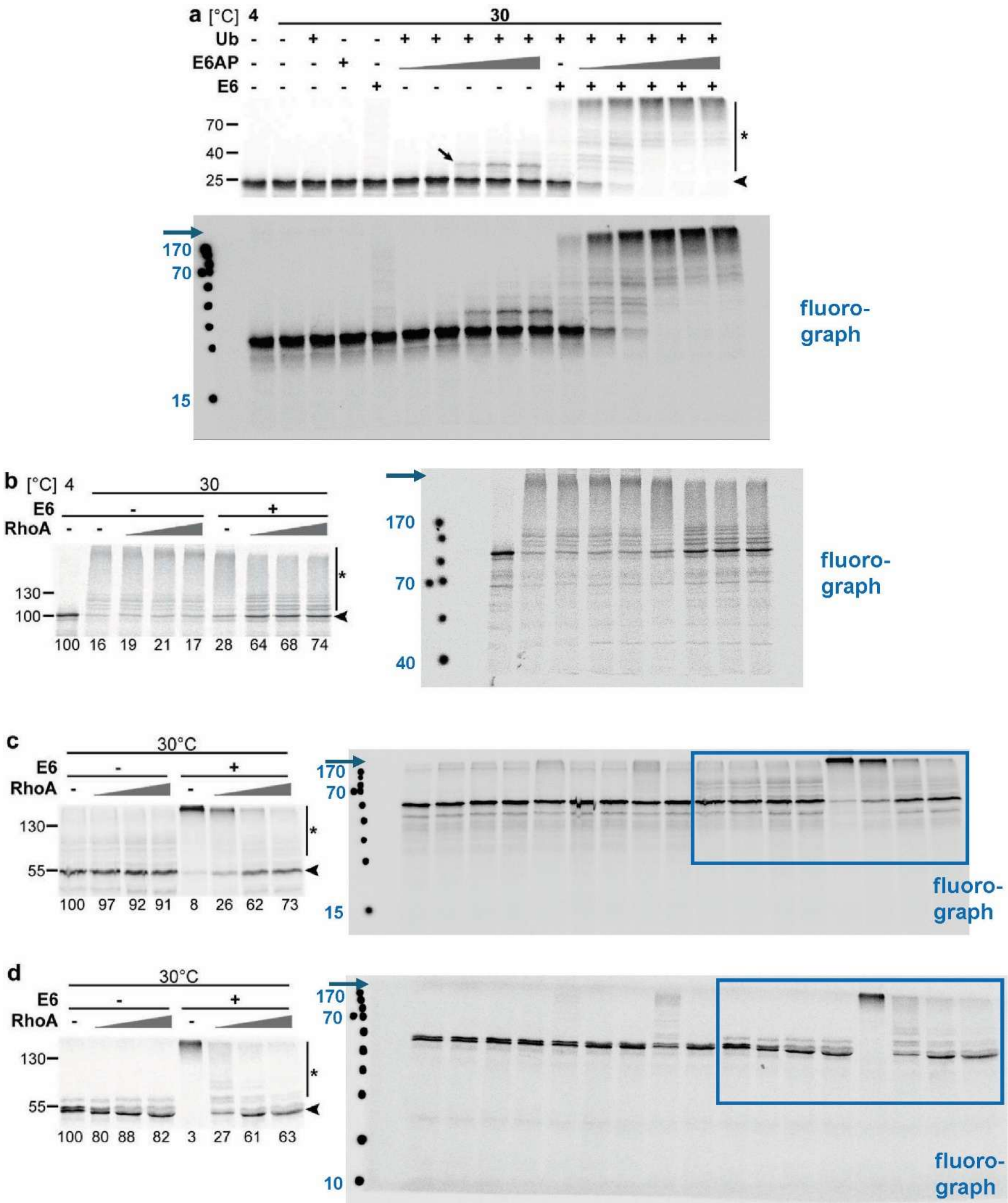

Fig. 1. Dots in the uncropped gels (fluorographs) indicate the running positions of molecular mass markers, with the 70 kDa marker indicated by two dots. Apparent molecular masses of the marker with the highest and with the lowest molecular mass are indicated. Arrow indicates the upper edge of the respective separating gel. Lanes of the gels shown in the actual figure are boxed, where appropriate. For further information, see main manuscript.

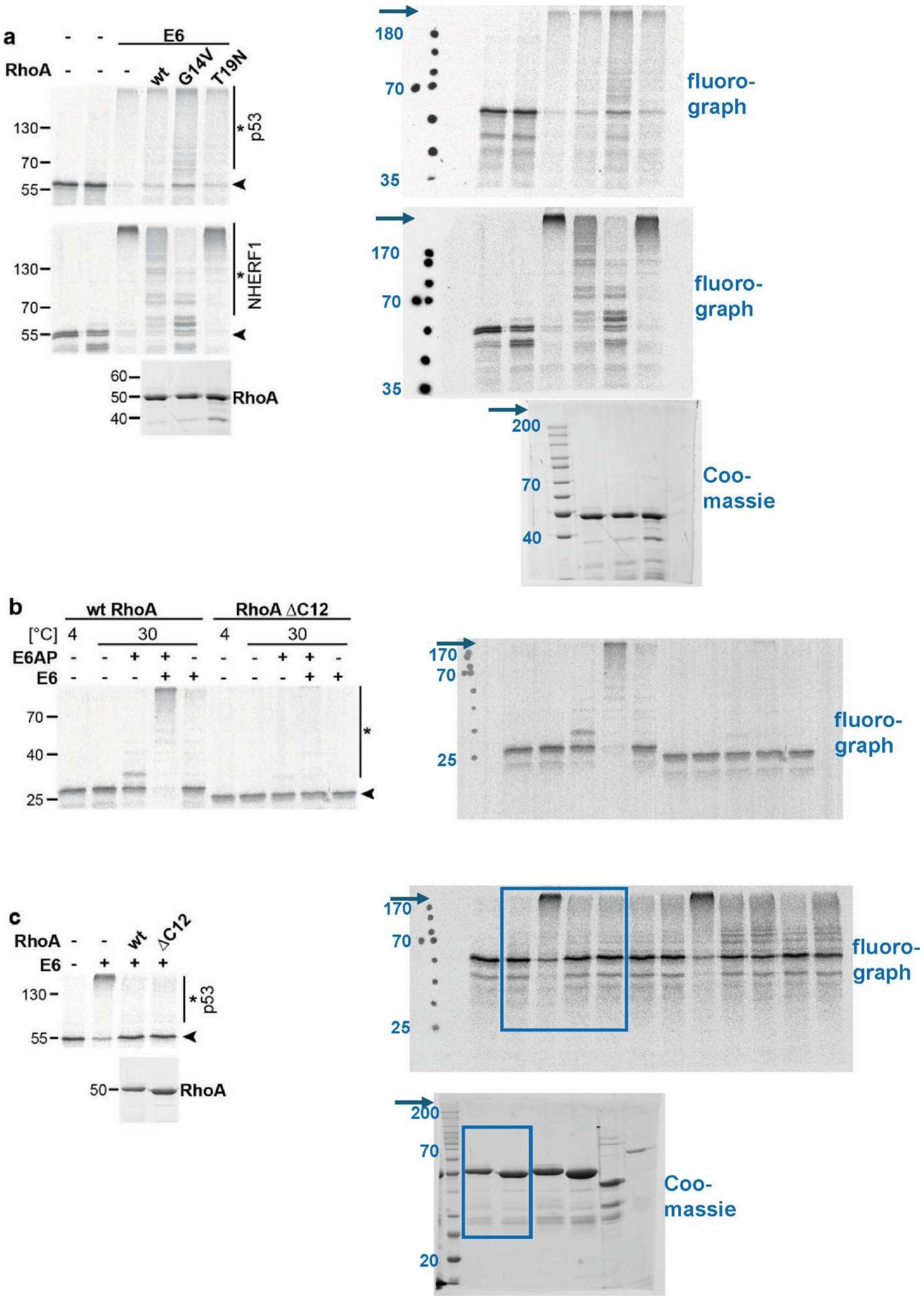

Fig. 2. Dots in the uncropped gels of fluorographs indicate the running positions of molecular mass markers, with the 70 kDa marker indicated by two dots. Apparent molecular masses of the marker with the highest and with the lowest molecular mass are indicated (note that for fluorographs a prestained marker was used, while for Coomassie-stained gels an unstained marker was used). Arrow indicates the upper edge of the respective separating gel. Lanes of the gels shown in the actual figure are boxed, where appropriate. For further information, see main manuscript.

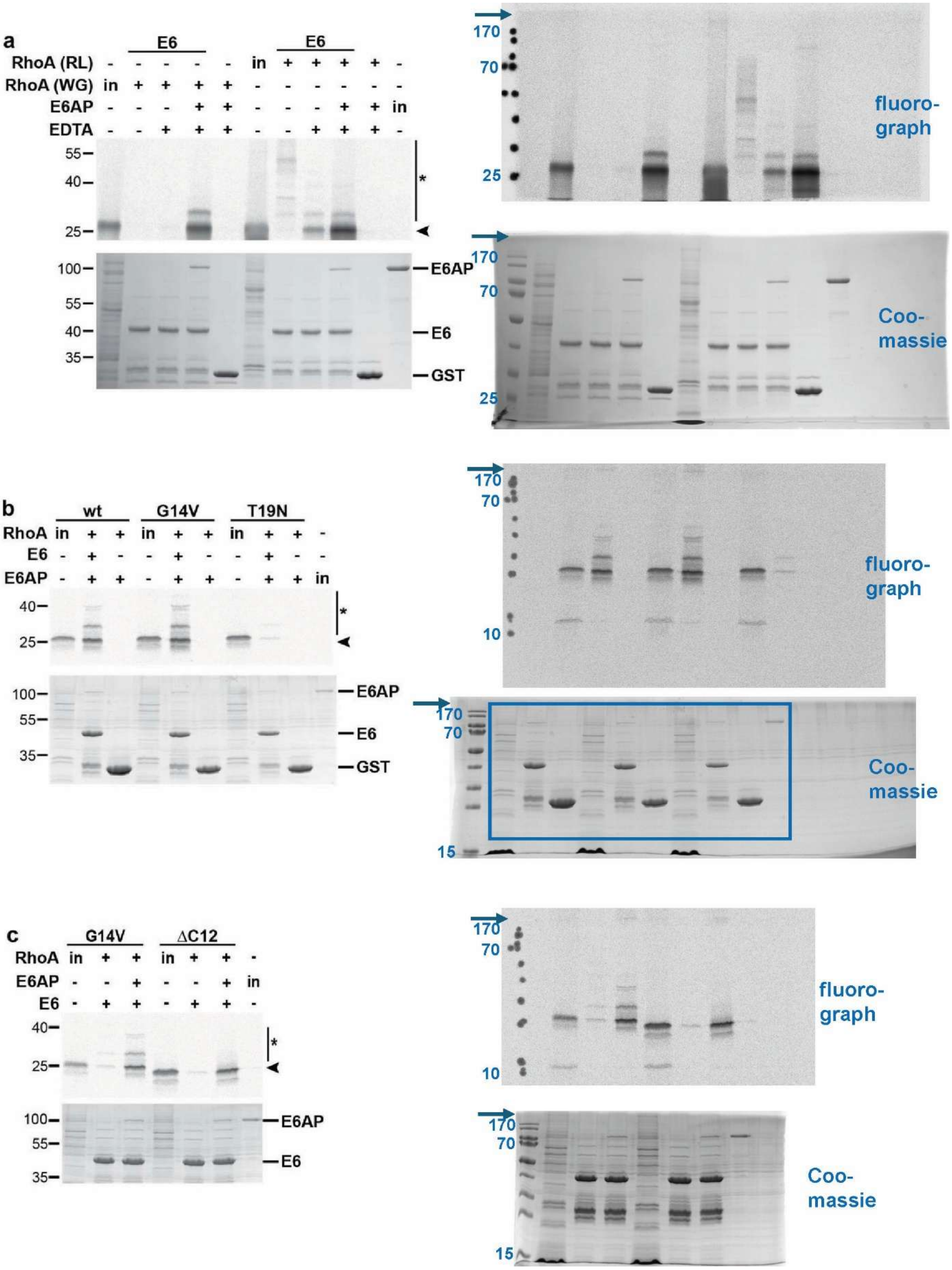

Fig. 3. Dots in the uncropped gels of fluorographs indicate the running positions of molecular mass markers, with the 70 kDa marker indicated by two dots. Apparent molecular masses of the marker with the highest and with the lowest molecular mass are indicated (note that for both fluorographs and Coomassie-stained gels, a prestained marker was used). Arrow indicates the upper edge of the respective separating gel. Lanes of the gels shown in the actual figure are boxed, where appropriate. For further information, see main manuscript.

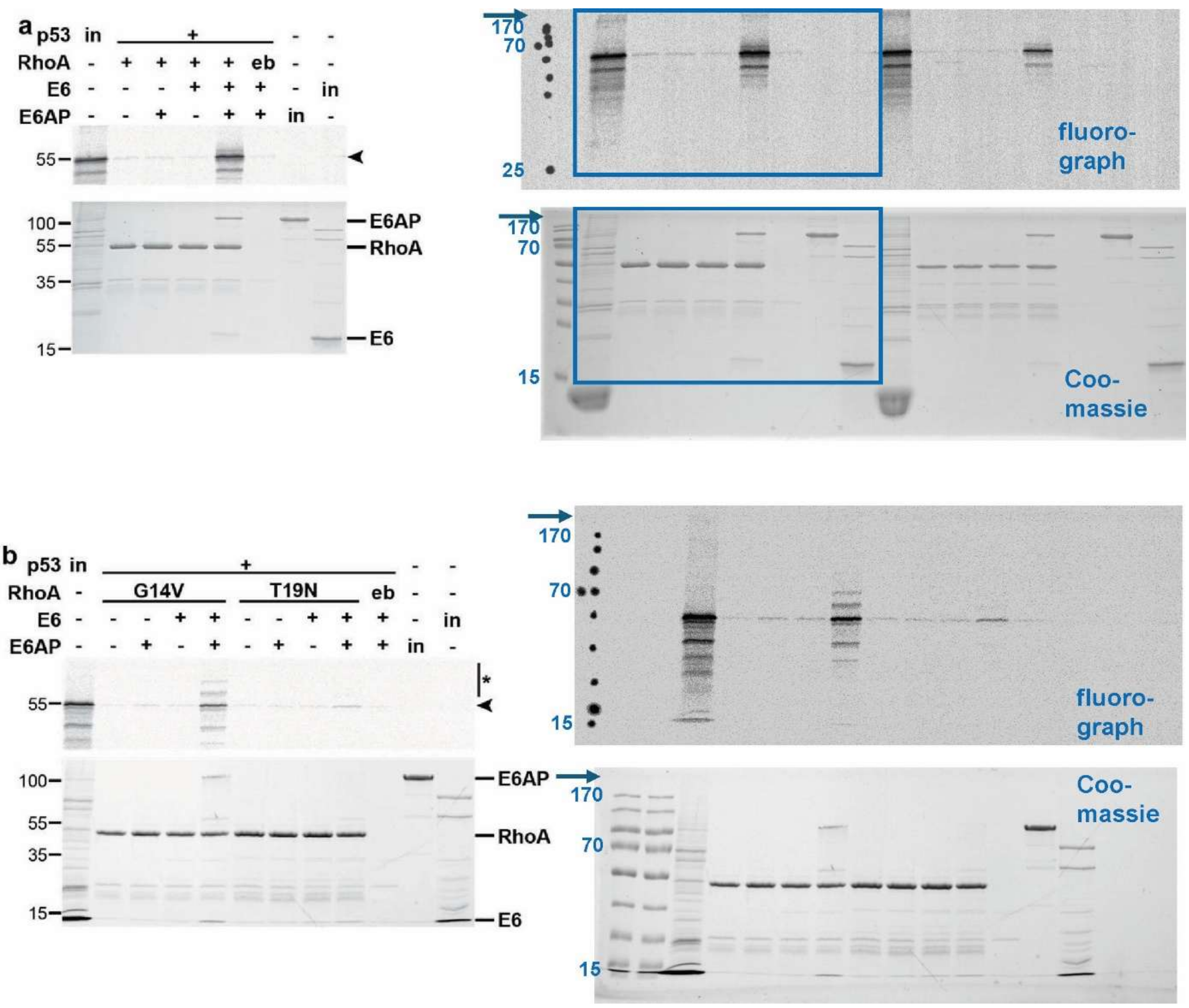

Fig. 4. Dots in the uncropped gels of fluorographs indicate the running positions of molecular mass markers, with the 70 kDa marker indicated by two dots. Apparent molecular masses of the marker with the highest and with the lowest molecular mass are indicated (note that for both fluorographs and Coomassie-stained gels, a prestained marker was used). Arrow indicates the upper edge of the respective separating gel. Lanes of the gels shown in the actual figure are boxed, where appropriate. For further information, see main manuscript.

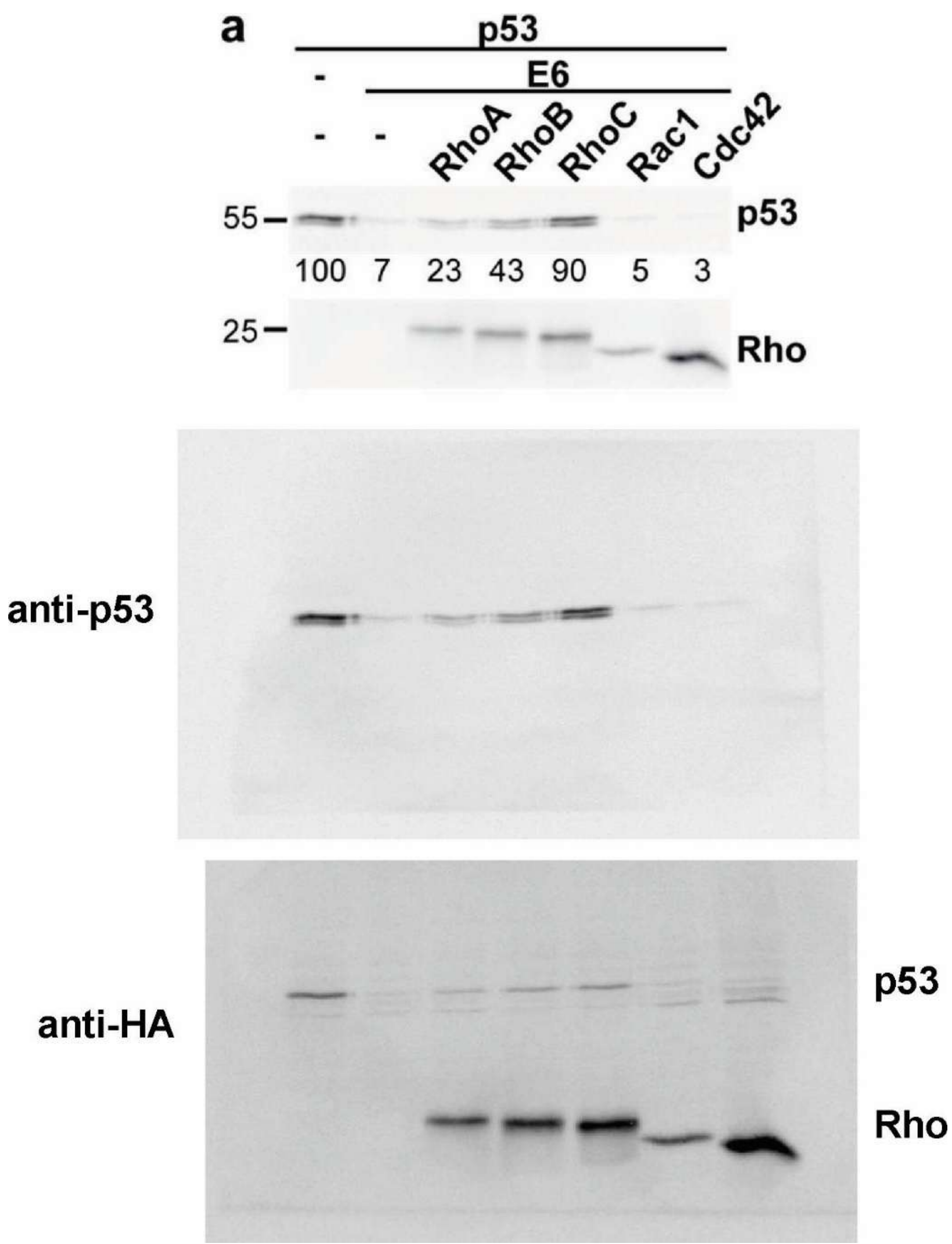

Fig. 6a. 24 h post-transfection, cell lysates were prepared, and transfection efficiency adjusted aliquots (Methods) were subjected to SDS-PAGE followed by western blot analysis. Upper blot was probed with an anti-p53 antibody (DO1). Lower blot was probed with the mouse monoclonal HA.11 antibody. Note that both p53 and the Rho variants were HA-tagged. For further information, see main manuscript.

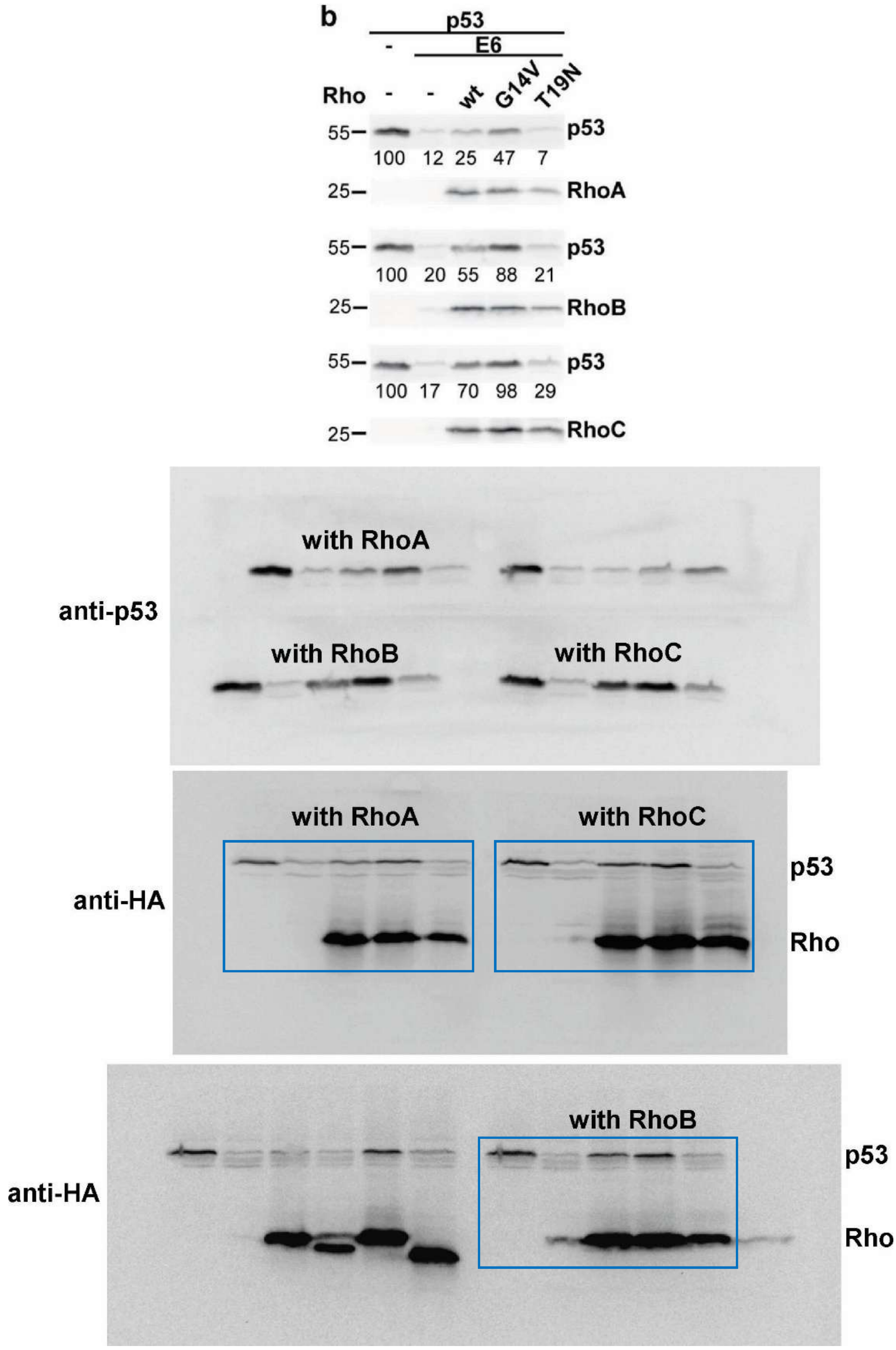

Fig. 6b. 24 h post-transfection, cell lysates were prepared, and transfection efficiency adjusted aliquots (Methods) were subjected to SDS-PAGE followed by western blot analysis. anti-p53, blots were probed with an anti-p53 antibody (DO1). anti-HA, blots were probed with the mouse monoclonal HA.11 antibody. Note that both p53 and the Rho variants were HA-tagged. Lanes of the gel shown in the actual figure are boxed. For further information, see main manuscript.

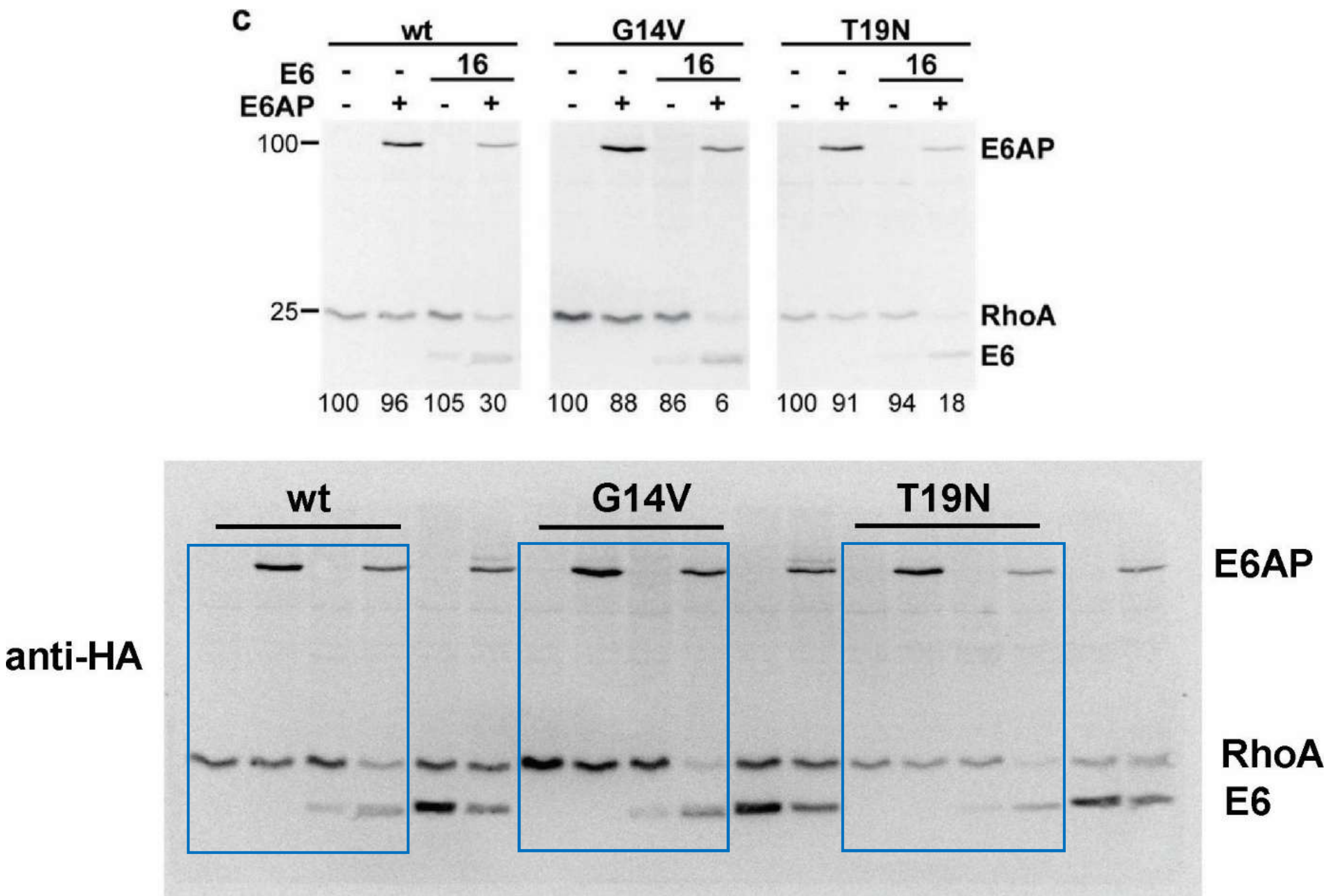

Fig. 6c. 24 h post-transfection, cell lysates were prepared, and transfection efficiency adjusted aliquots (Methods) were subjected to SDS-PAGE followed by western blot analysis using the mouse monoclonal HA.11 antibody. Note that E6, E6AP, and the RhoA variants were HA-tagged. Lanes of the gel shown in the actual figure are boxed. For further information, see main manuscript.

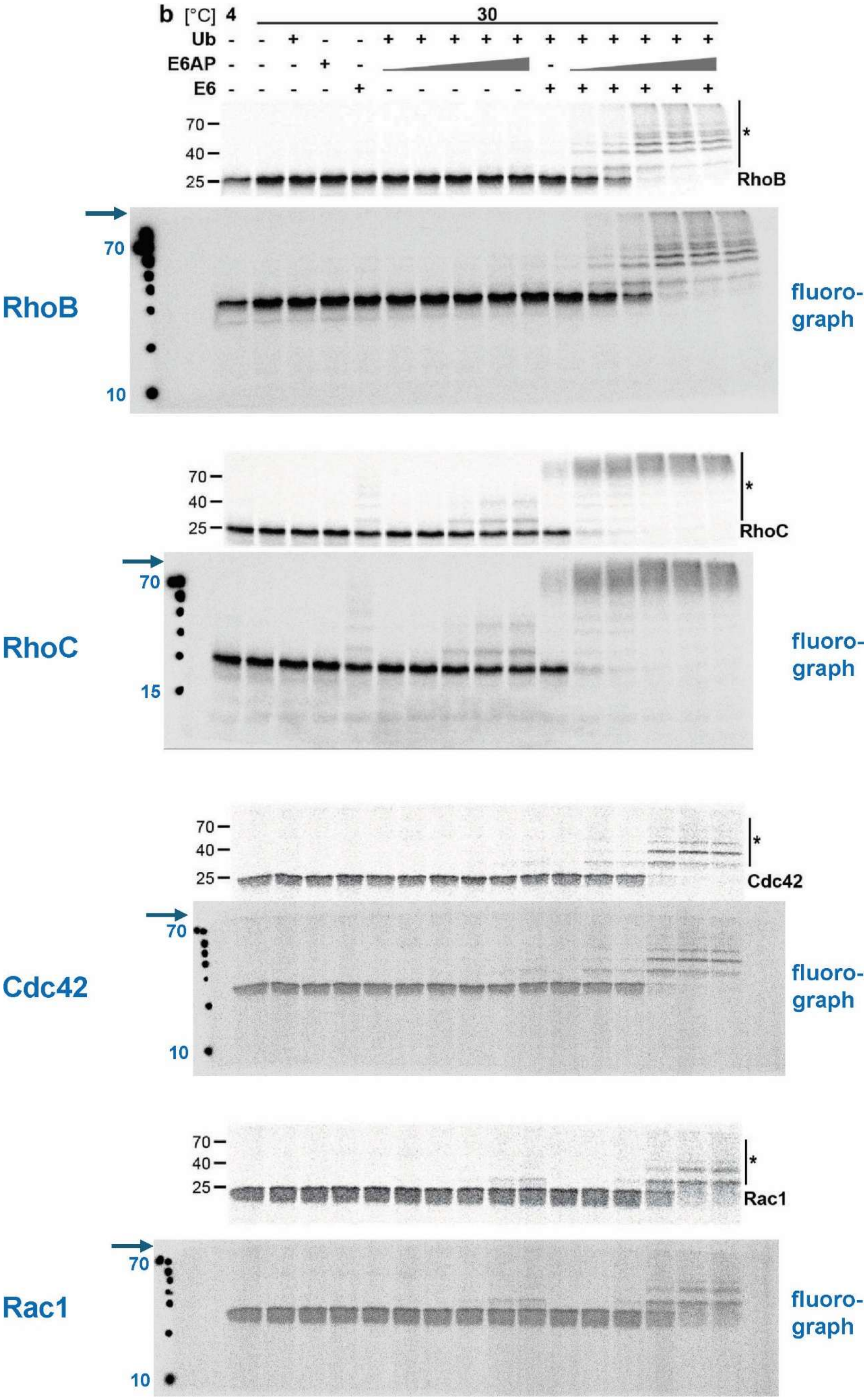

Supplementary Fig. 1b. Dots in the uncropped gels (fluorographs) indicate the running positions of molecular mass markers, with the 70 kDa marker indicated by two dots. Apparent molecular mass of the marker with the lowest molecular mass is indicated. Arrow indicates the upper edge of the respective separating gel. For further information, see main manuscript.

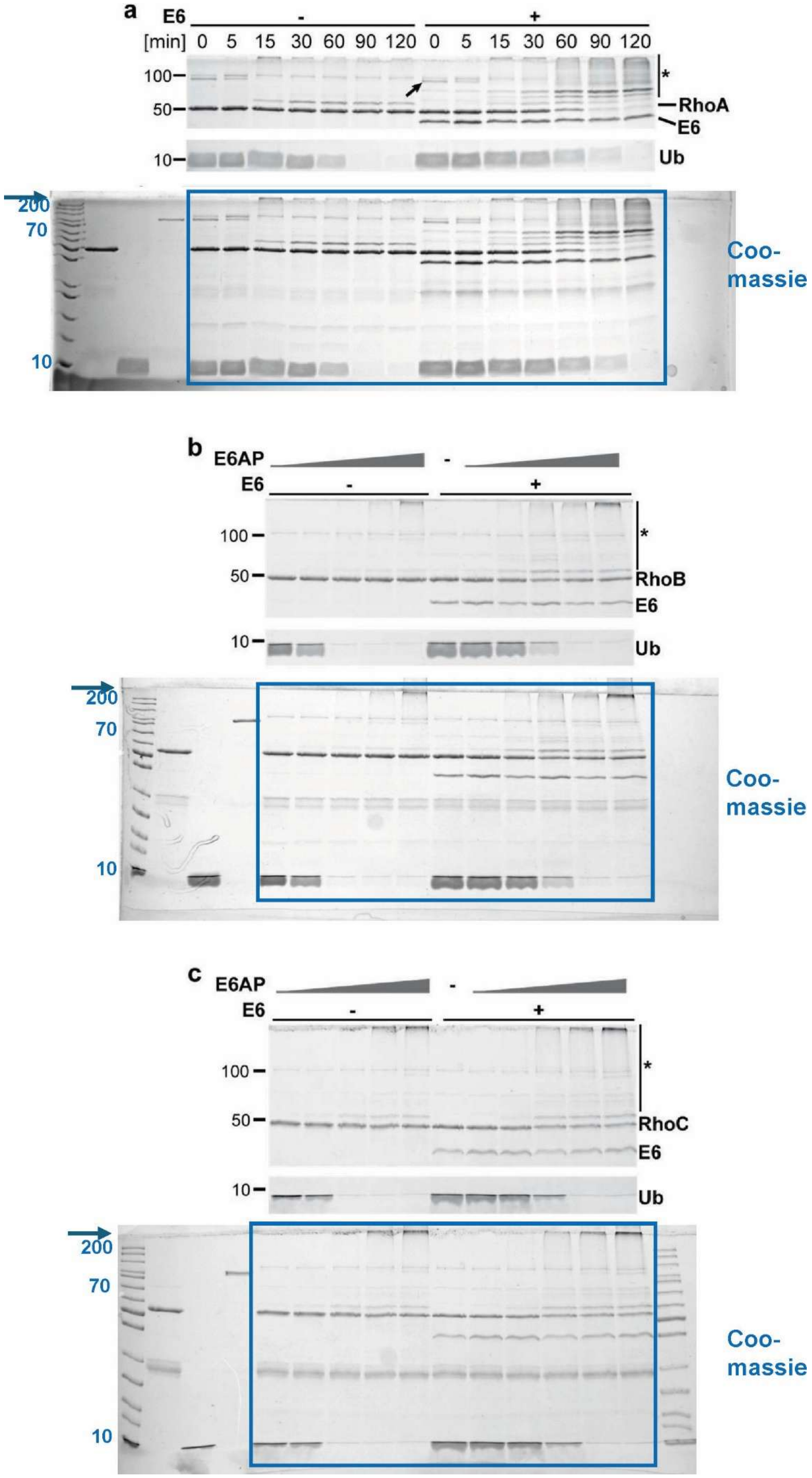

Supplementary Fig. 2a-c. Apparent molecular masses of the 70 kDa marker and the marker with the highest and with the lowest molecular mass are indicated (Coomassie-stained gels). Arrow indicates the upper edge of the respective separating gel. Lanes of the gels shown in the actual figure are boxed, where appropriate. For further information, see main manuscript.

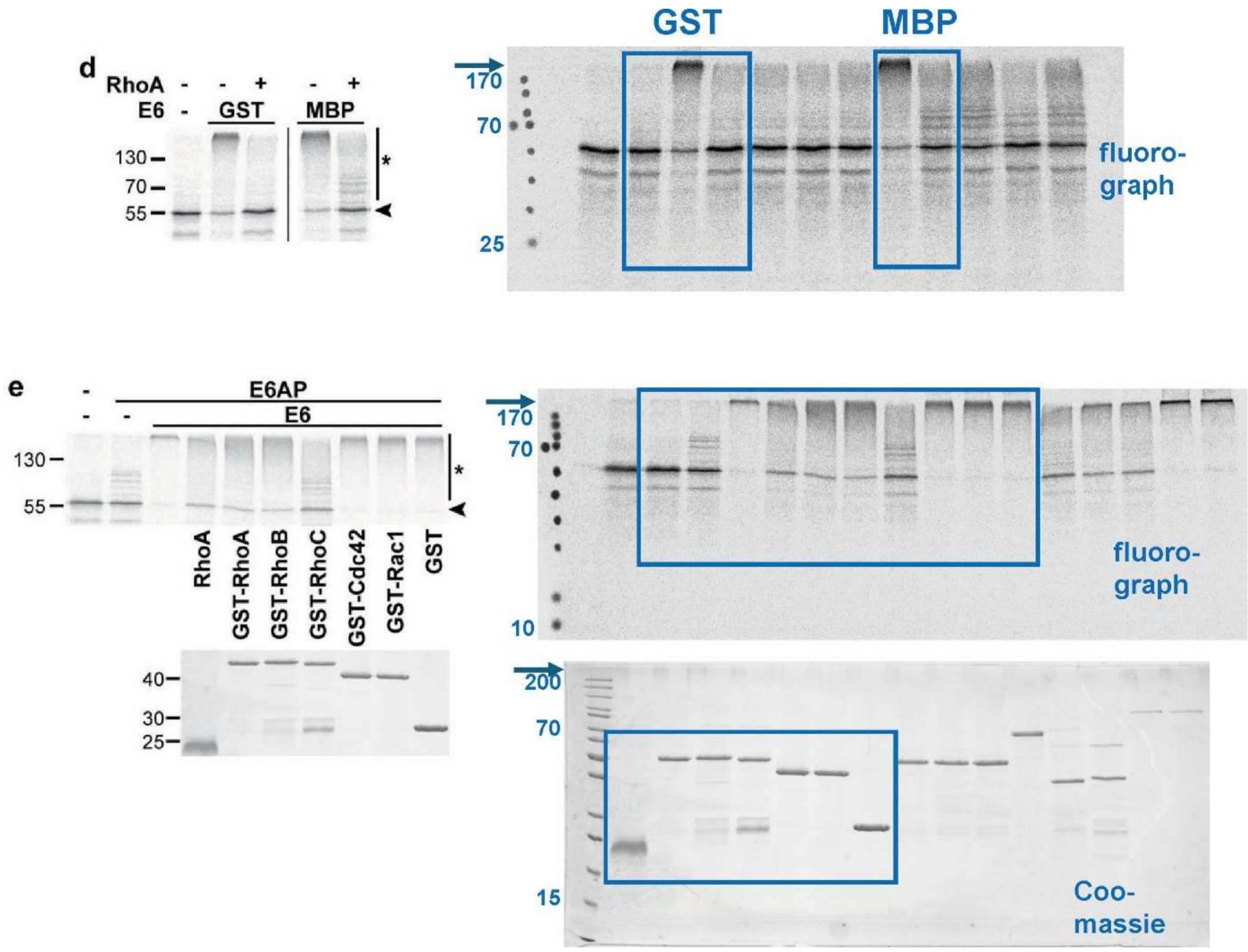

Supplementary Fig. 2d,e. Dots in the uncropped gels of fluorographs indicate the running positions of molecular mass markers, with the 70 kDa marker indicated by two dots. Apparent molecular masses of the marker with the highest and with the lowest molecular mass are indicated (note that for fluorographs a prestained marker was used, while for the Coomassie-stained gel an unstained marker was used). Arrow indicates the upper edge of the respective separating gel. Lanes of the gels shown in the actual figure are boxed, where appropriate. For further information, see main manuscript.

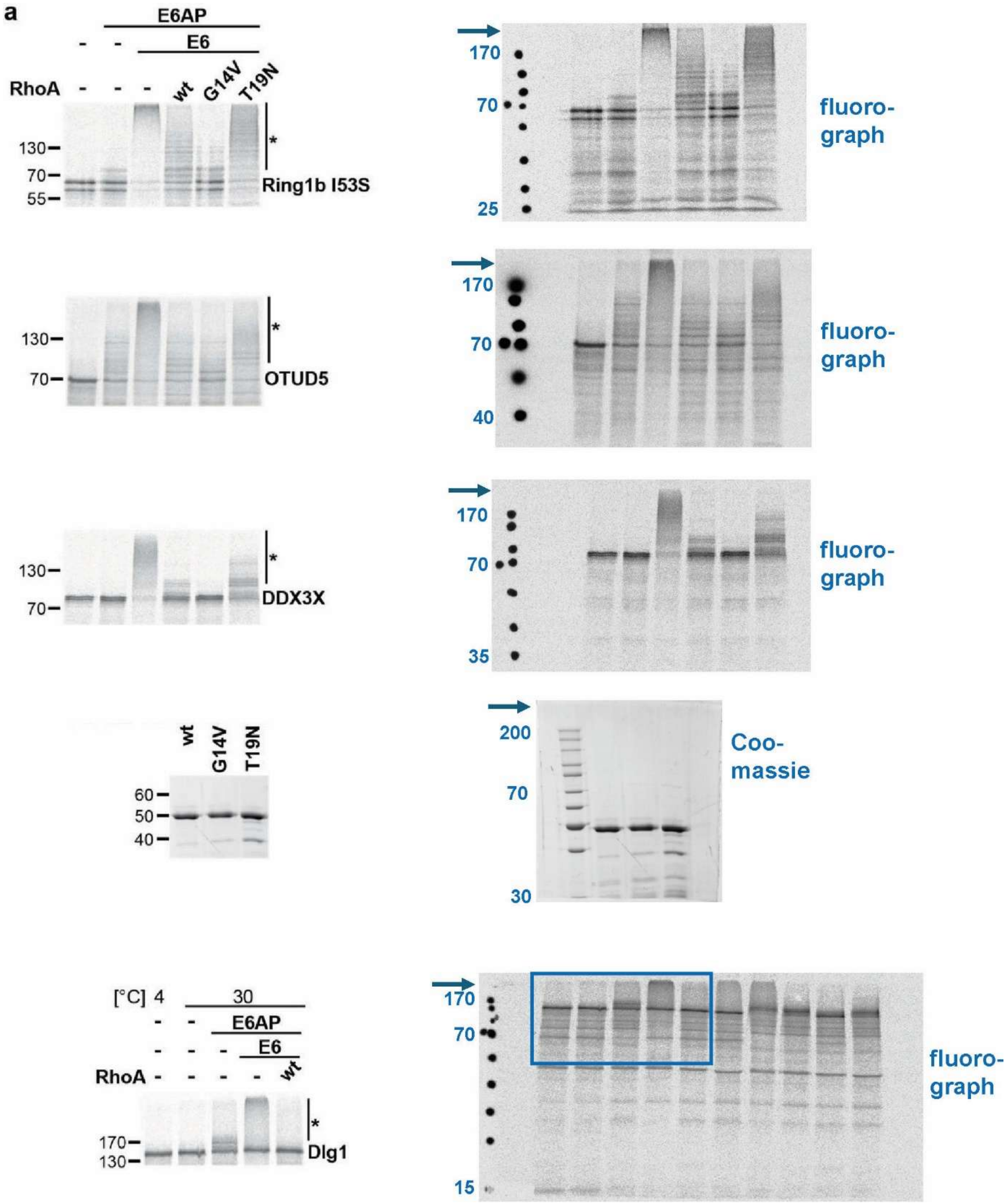

Supplementary Fig. 3a. Dots in the uncropped gels of fluorographs indicate the running positions of molecular mass markers, with the 70 kDa marker indicated by two dots. Apparent molecular masses of the marker with the highest and with the lowest molecular mass are indicated (note that for fluorographs a prestained marker was used, while for the Coomassie-stained gel an unstained marker was used). Arrow indicates the upper edge of the respective separating gel. Lanes of the gels shown in the actual figure are boxed, where appropriate. For further information, see main manuscript.

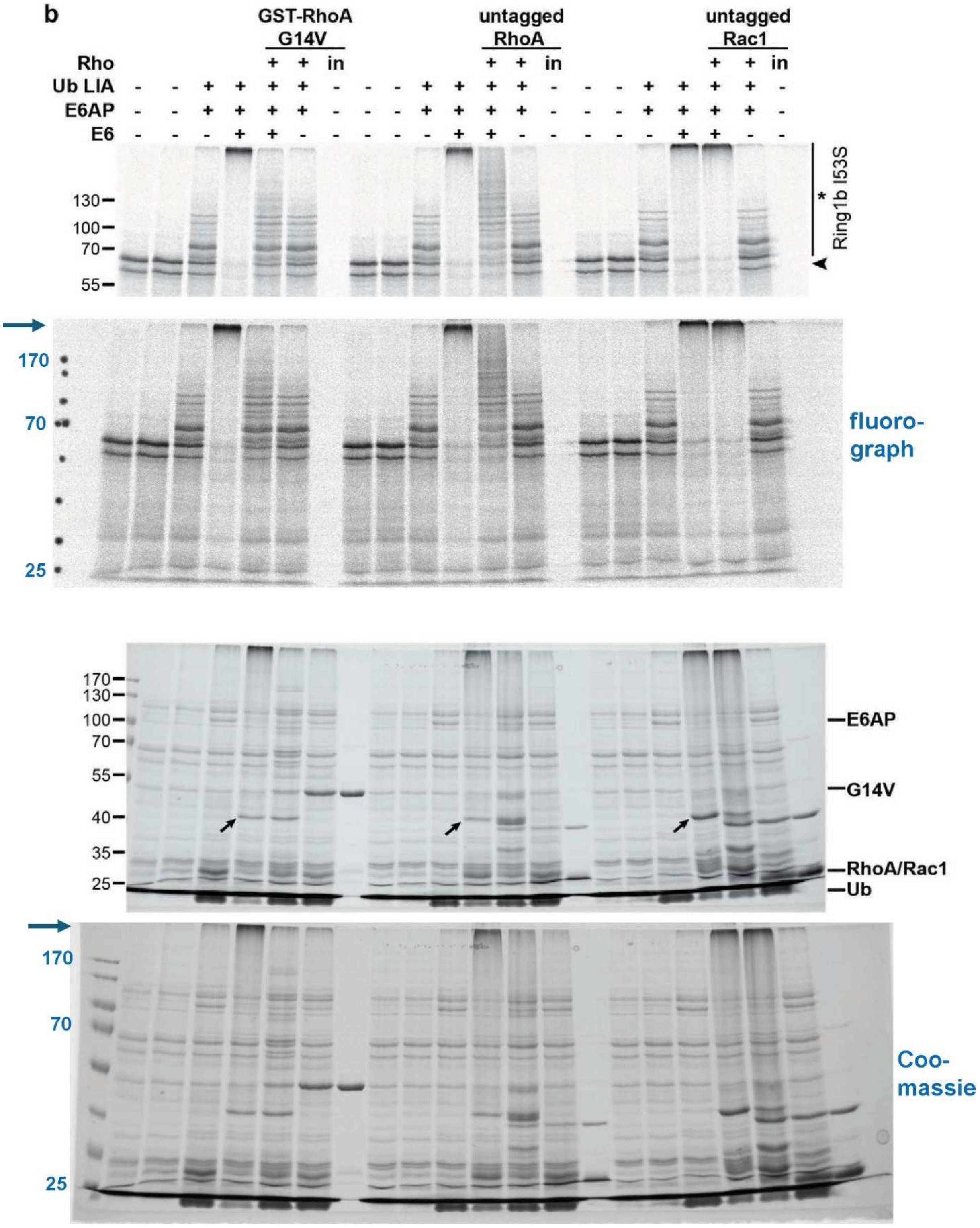

Supplementary Fig. 3b. Dots in the uncropped gel of the fluorograph indicate the running positions of molecular mass markers, with the 70 kDa marker indicated by two dots. Apparent molecular masses of the marker with the highest and with the lowest molecular mass are indicated (note that for both the fluorograph and the Coomassie-stained gel, a prestained marker was used). Arrow indicates the upper edge of the respective separating gel. For further information, see main manuscript.

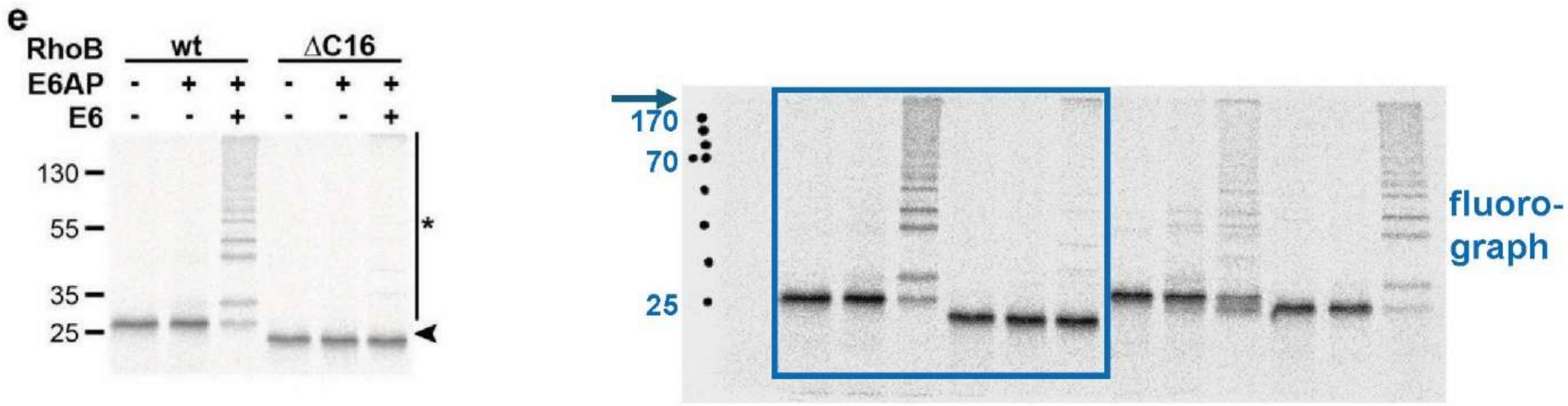

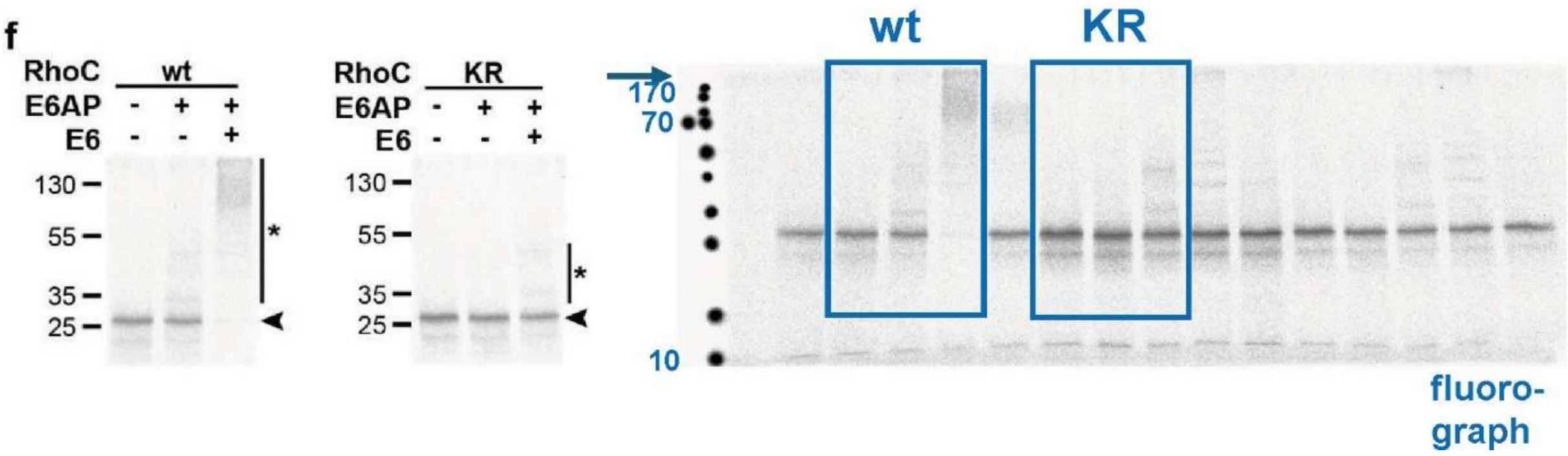

Supplementary Fig. 3e,f. Dots in the uncropped gels (fluorographs) indicate the running positions of molecular mass markers, with the 70 kDa marker indicated by two dots. Apparent molecular masses of the marker with the highest and with the lowest molecular mass are indicated. Arrow indicates the upper edge of the respective separating gel. Lanes of the gels shown in the actual figure are boxed, where appropriate. For further information, see main manuscript.

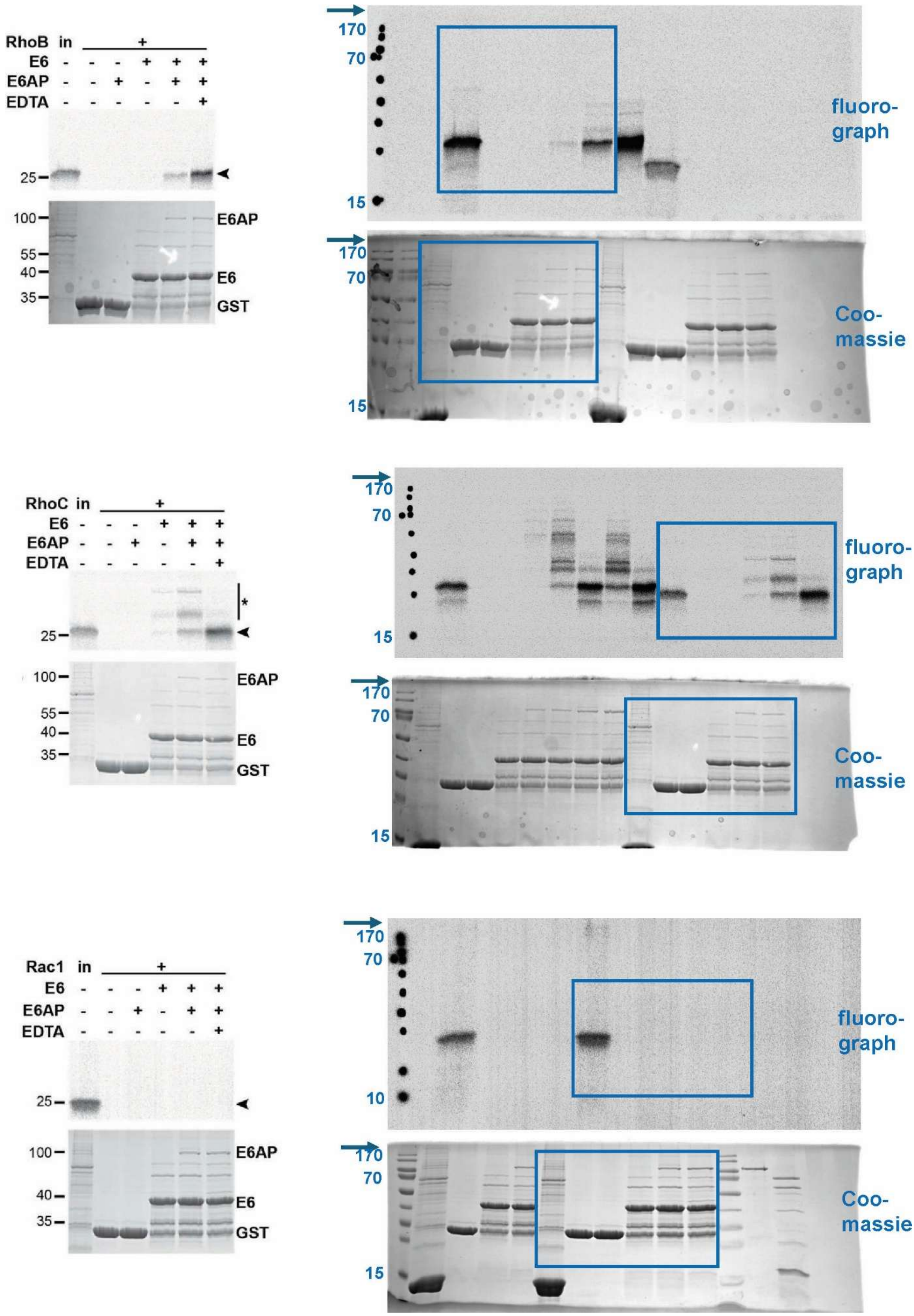

Supplementary Fig. 4. Dots in the uncropped gels of fluorographs indicate the running positions of molecular mass markers, with the 70 kDa marker indicated by two dots. Apparent molecular masses of the marker with the highest and with the lowest molecular mass are indicated (note that for both fluorographs and Coomassie-stained gels, a prestained marker was used). Arrow indicates the upper edge of the respective separating gel. Lanes of the gels shown in the actual figure are boxed, where appropriate. For further information, see main manuscript.

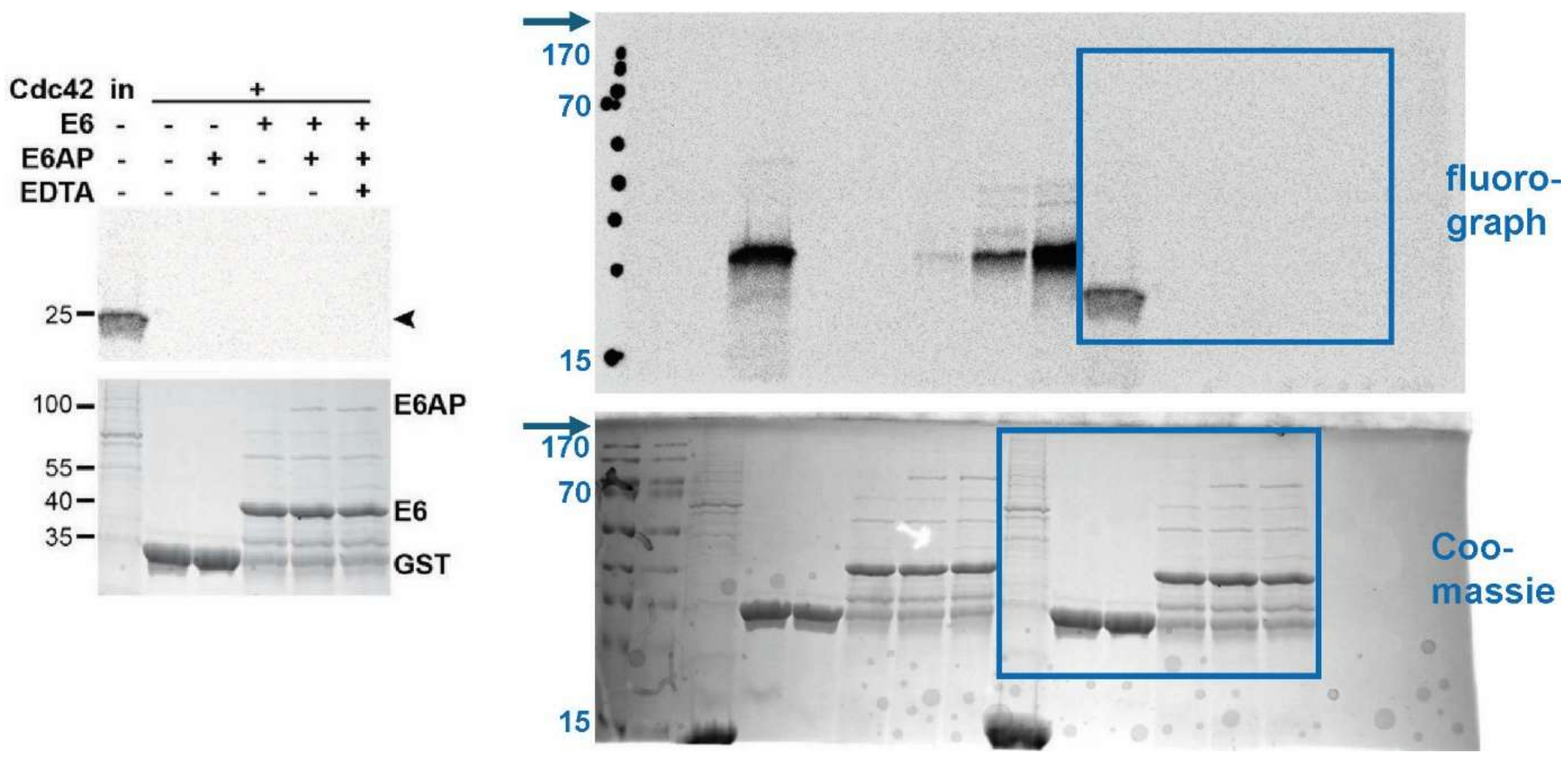

Supplementary Fig. 4. Dots in the uncropped gel of the fluorograph indicate the running positions of molecular mass markers, with the 70 kDa marker indicated by two dots. Apparent molecular masses of the marker with the highest and with the lowest molecular mass are indicated (note that for both the fluorograph and the Coomassie-stained gel, a prestained marker was used). Arrow indicates the upper edge of the respective separating gel. Lanes of the gels shown in the actual figure are boxed, where appropriate. For further information, see main manuscript.

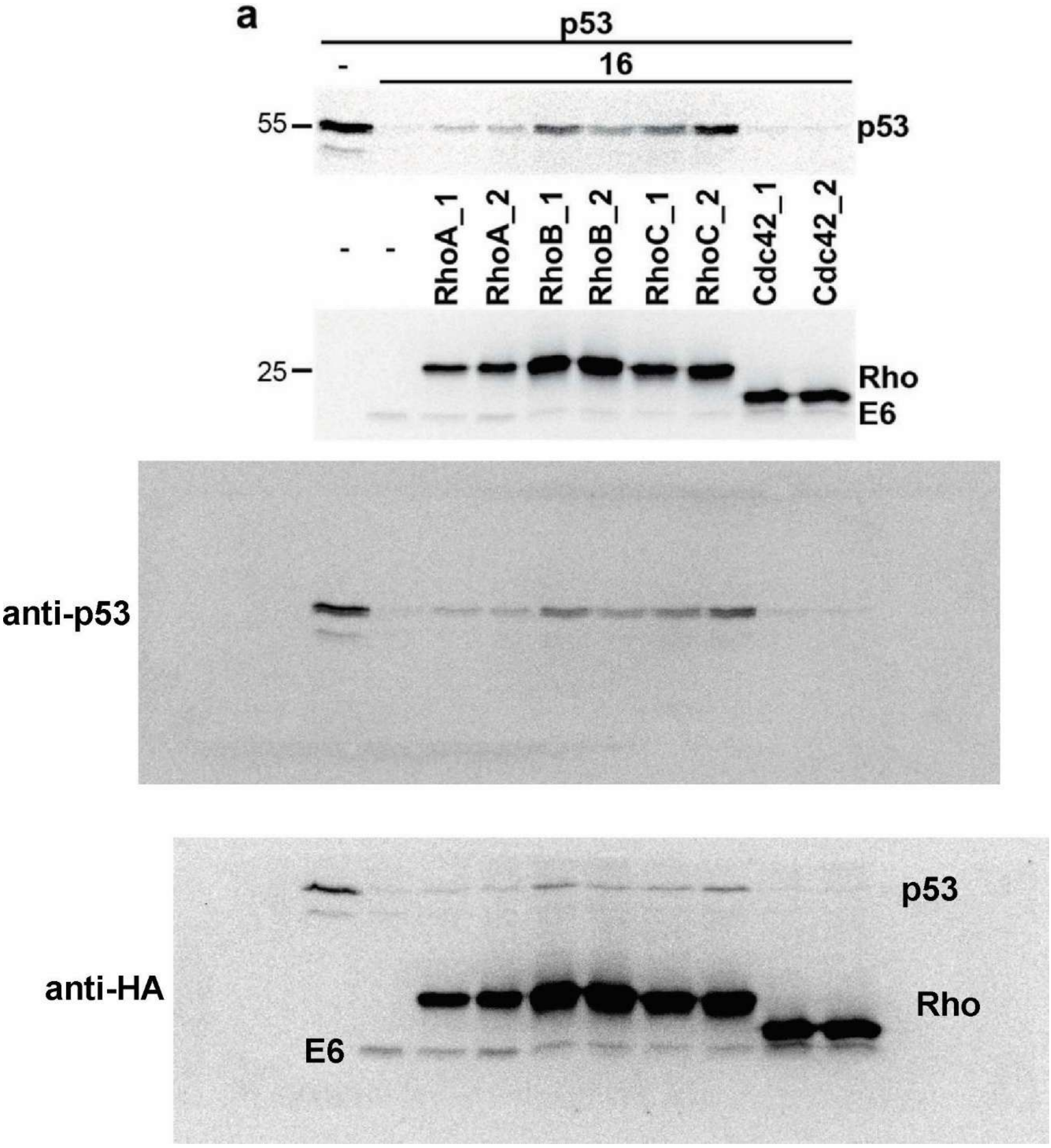

Suppl. Fig. 7a. 24 h post-transfection, cell lysates were prepared, and transfection efficiency adjusted aliquots (Methods) were subjected to SDS-PAGE followed by western blot analysis.anti-p53, blot was probed with an anti-p53 antibody (DO1). anti-HA, blot was probed with the mouse monoclonal HA.11 antibody. Note that both p53, the Rho variants, and E6 were HA-tagged. For further information, see main manuscript.

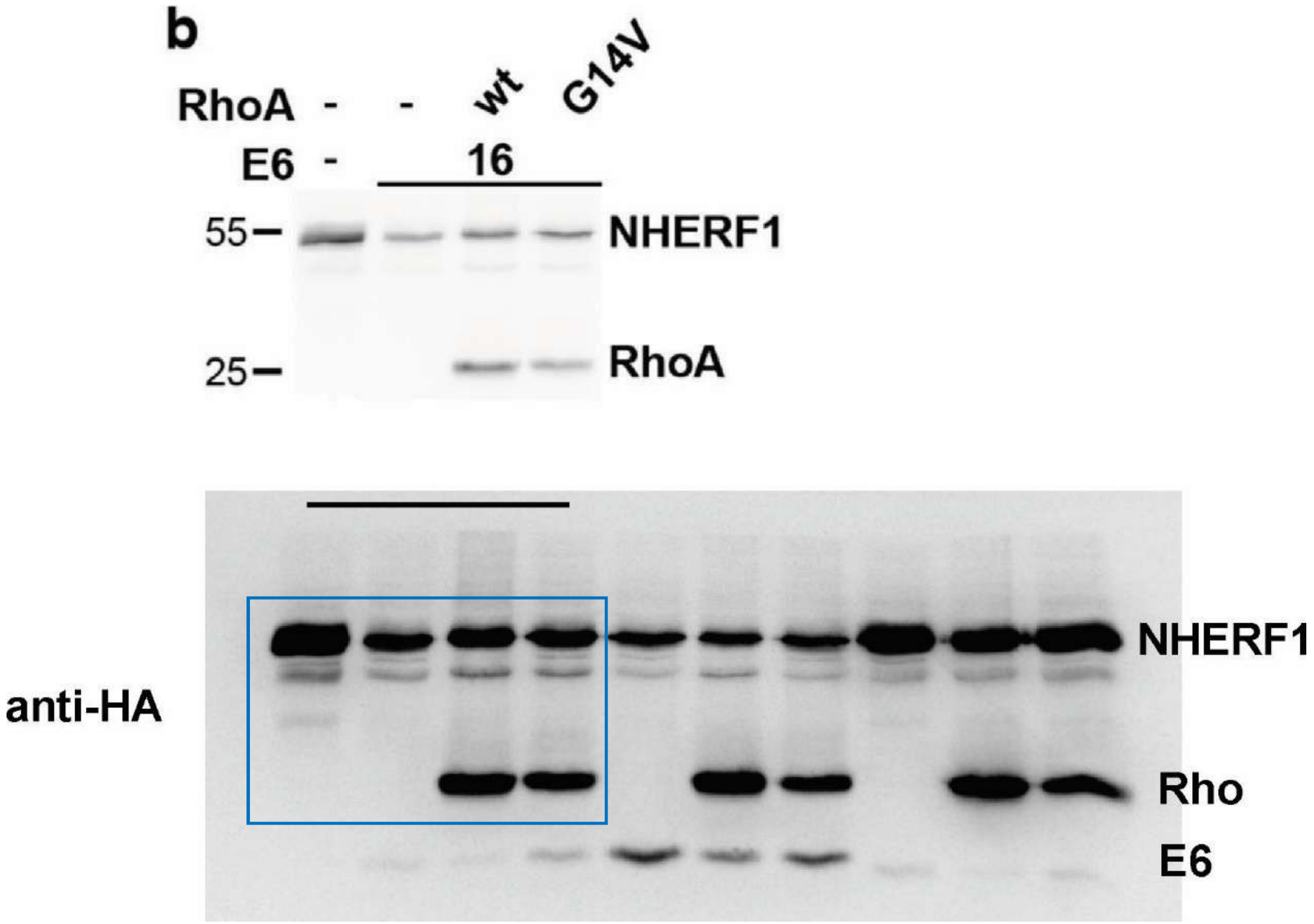

Suppl. Fig. 7b. 24 h post-transfection, cell lysates were prepared, and transfection efficiency adjusted aliquots (Methods) were subjected to SDS-PAGE followed by western blot analysis using the mouse monoclonal HA.11 antibody. Note that NHERF1, the RhoA variants, and E6 were HA-tagged. Lanes of the gel shown in the actual figure are boxed. For further information, see main manuscript.

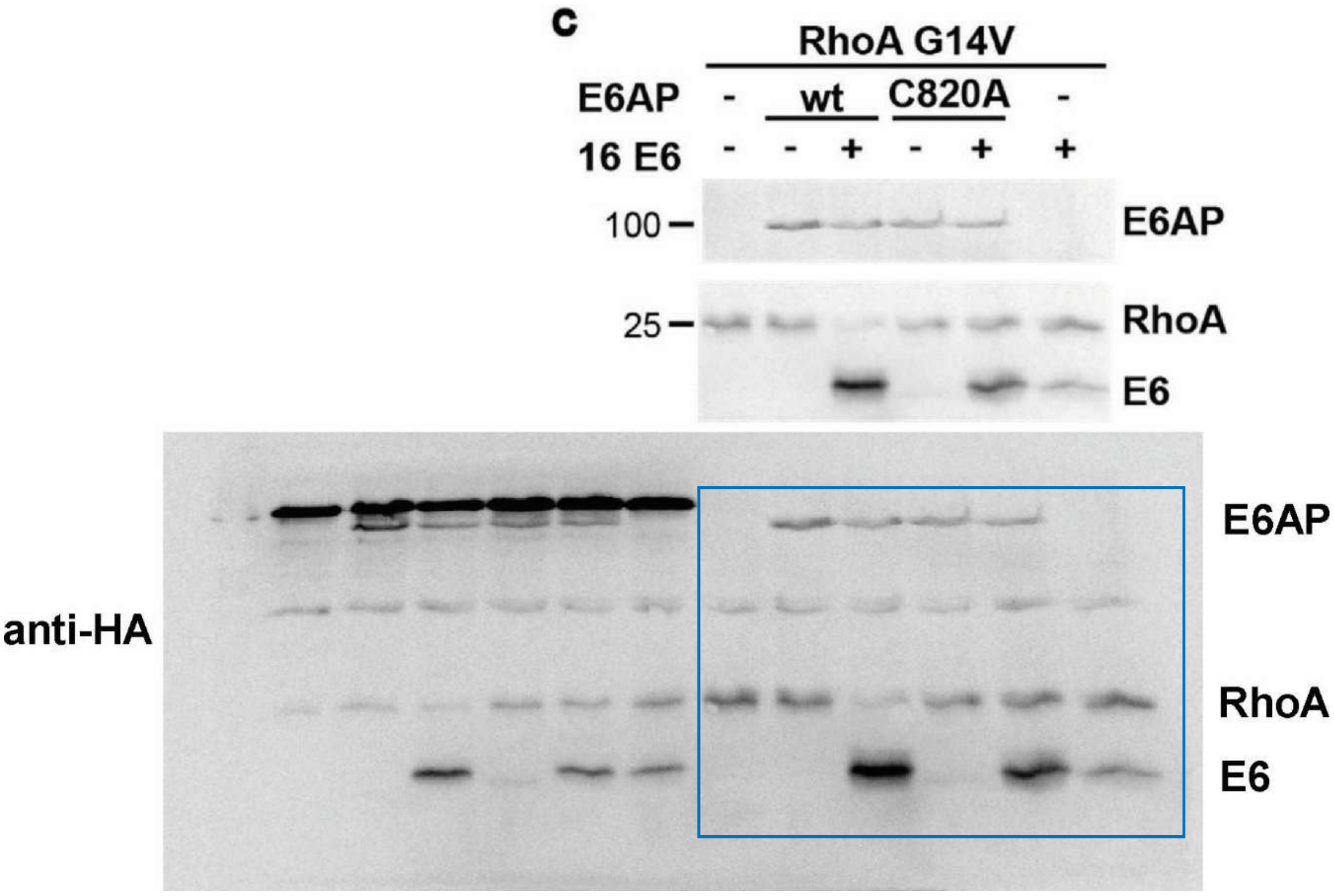

Suppl. Fig. 7c. 24 h post-transfection, cell lysates were prepared, and transfection efficiency adjusted aliquots (Methods) were subjected to SDS-PAGE followed by western blot analysis using the mouse monoclonal HA.11 antibody. Note that E6AP (wt), a catalytically inactive E6AP variant (C820A), E6, and RhoA were HA-tagged. Lanes of the gel shown in the actual figure are boxed. For further information, see main manuscript.

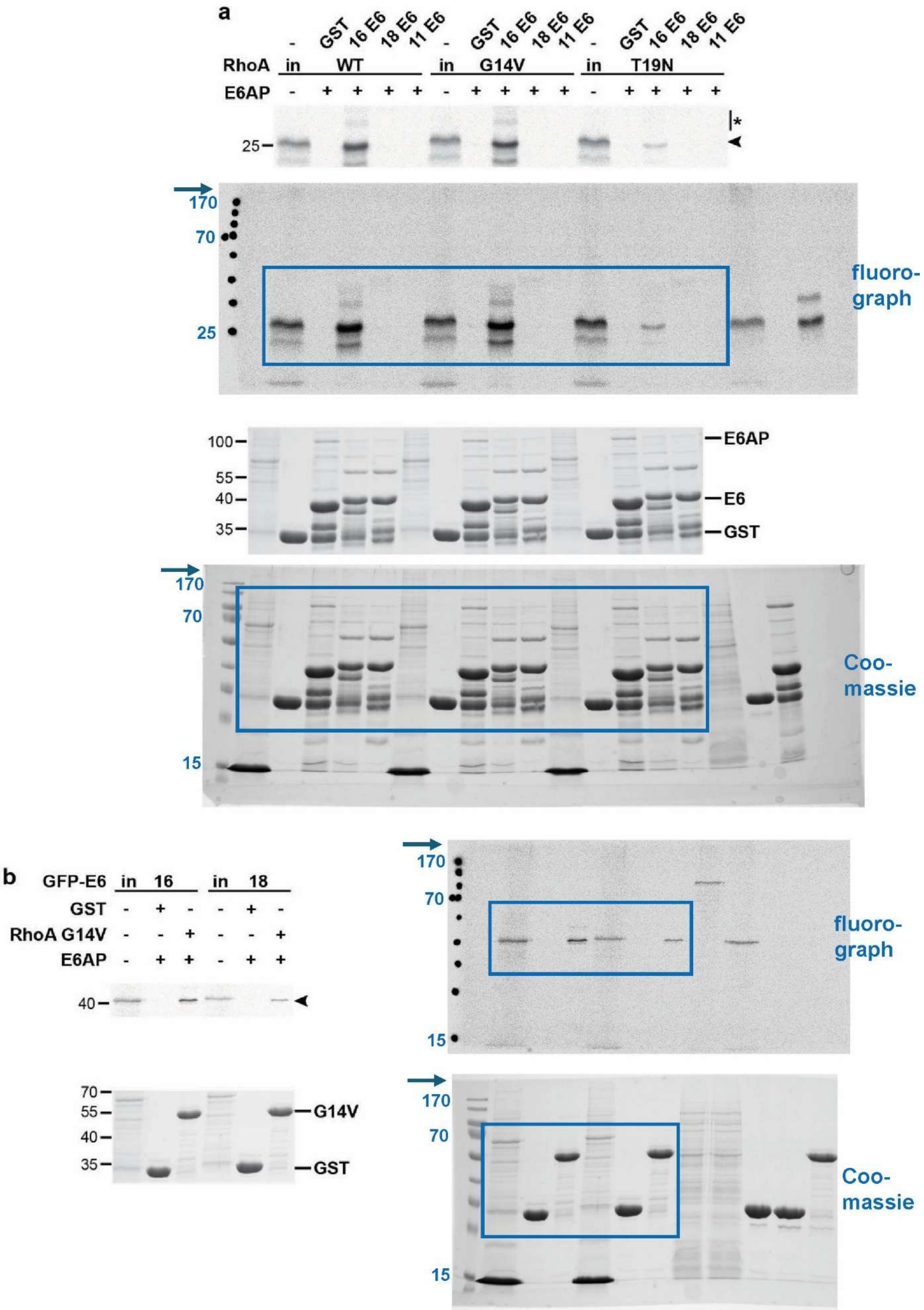

Supplementary Fig. 8a,b. Dots in the uncropped gels of fluorographs indicate the running position of molecular mass markers, with the 70 kDa marker indicated by two dots. Apparent molecular masses of the marker with the highest and with the lowest molecular mass are indicated (note that for both fluorographs and Coomassie-stained gels, a prestained marker was used). Arrow indicates the upper edge of the respective separating gel. Lanes of the gels shown in the actual figure are boxed, where appropriate. For further information, see main manuscript.

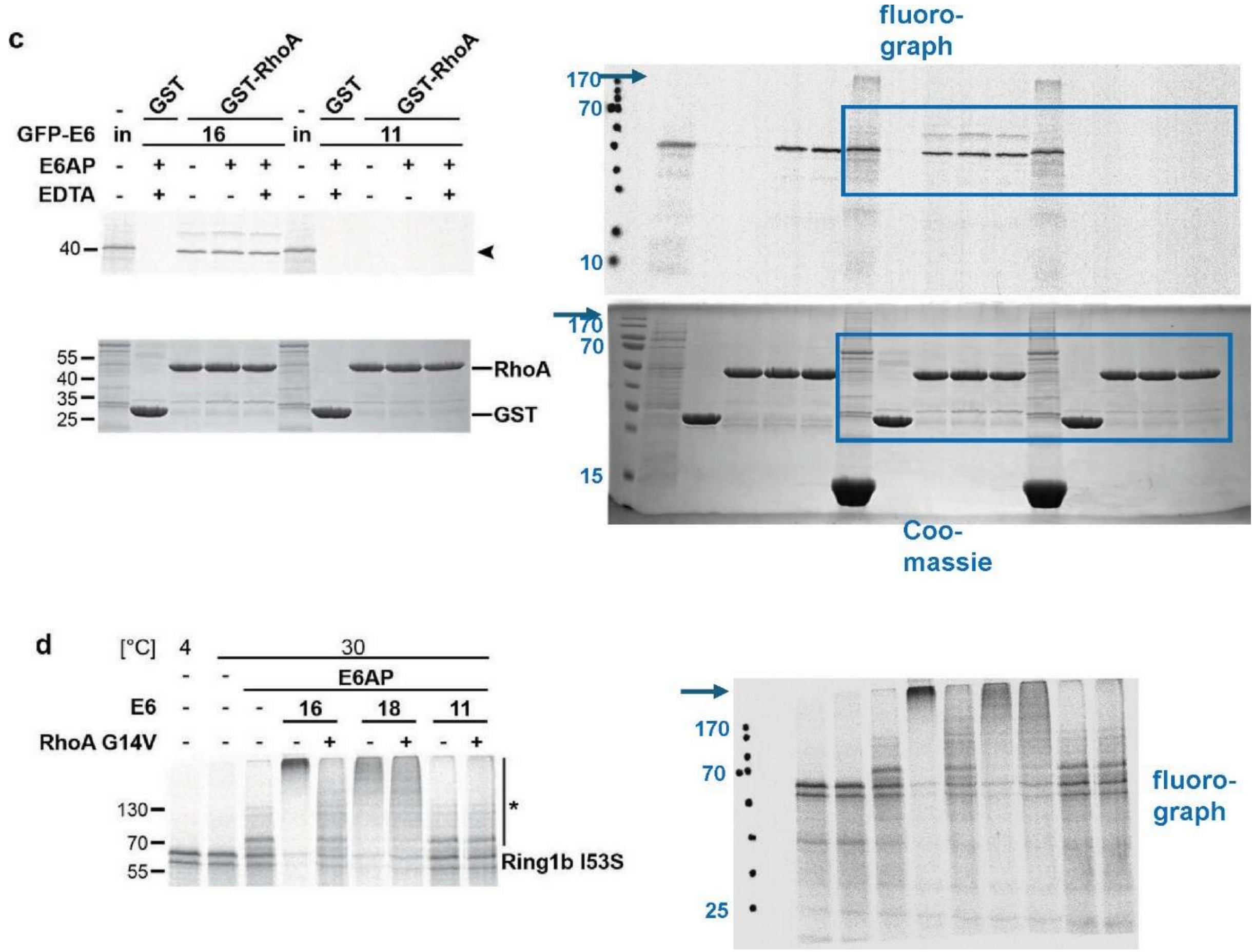

Supplementary Fig. 8c,d. Dots in the uncropped gels of fluorographs indicate the running position of molecular mass markers, with the 70 kDa marker indicated by two dots. Apparent molecular masses of the marker with the highest and with the lowest molecular mass are indicated (note that for both fluorographs and the Coomassie-stained gel, a prestained marker was used). Arrow indicates the upper edge of the respective separating gel. Lanes of the gels shown in the actual figure are boxed, where appropriate. For further information, see main manuscript.
